# Rate and Predictors of Mucosal Healing in Patients with Inflammatory Bowel Disease Treated with Anti-TNF-Alpha Antibodies

**DOI:** 10.1371/journal.pone.0099293

**Published:** 2014-06-16

**Authors:** Florian Beigel, Matthias Deml, Fabian Schnitzler, Simone Breiteneicher, Burkhard Göke, Thomas Ochsenkühn, Stephan Brand

**Affiliations:** 1 Department of Medicine II, University Hospital Munich-Grosshadern, Ludwig-Maximilians-University, Munich, Germany; 2 Isarmedizin Zentrum, Munich, Germany; University of South Carolina School of Medicine, United States of America

## Abstract

**Objective:**

Mucosal healing (MH) is an important treatment goal in patients with inflammatory bowel disease (IBD), but factors predicting MH under medical therapy are largely unknown. In this study, we aimed to characterize predictive factors for MH in anti-TNF-alpha antibody-treated IBD patients.

**Methods:**

We retrospectively analyzed 248 IBD patients (61.3% CD, 38.7% UC) treated with anti-TNF-alpha antibodies (infliximab and/or adalimumab) for MH, defined as macroscopic absence of inflammatory lesions (Mayo endoscopy score 0 or SES-CD score 0) in colonoscopies which were analyzed before and after initiation of an anti-TNF-alpha antibody treatment.

**Results:**

In patients treated with only one anti-TNF-alpha antibody (“TNF1 group”, n = 202), 56 patients (27.7%) achieved complete MH at follow-up colonoscopy (median overall follow-up time: 63 months). In a second cohort (n = 46), which comprised patients who were consecutively treated with two anti-TNF-alpha antibodies (“TNF2 group”), 13 patients (28.3%) achieved complete MH (median overall follow-up time: 64.5 months). Compared to patients without MH, CRP values at follow-up colonoscopy were significantly lower in patients with MH (TNF1 group: p = 8.35×10^−5^; TNF2 group: p = 0.002). Multivariate analyses confirmed CRP at follow-up colonoscopy as predictor for MH in the TNF1 group (p = 0.012). Overall need for surgery was lower in patients with MH (TNF1 group: p = 0.01; TNF2 group: p = 0.03).

**Conclusions:**

We identified low serum CRP level at follow-up colonoscopy as predictor for MH, while MH was an excellent negative predictor for the need for surgery.

## Introduction

Treatment of patients with inflammatory bowel disease (IBD) has been focused but is also currently focused on symptomatic relief and clinical improvement. However, since the course of IBD may progress from an inflammatory to a stricturing and penetrating type of disease with a high rate of bowel surgery (up to 80% in Crohn's disease (CD)) [1], [2], early and sufficient treatment strategies to protect the mucosal integrity and therefore prevent disease progression are warranted. Colonoscopy is the gold standard for diagnosing mucosal injury in IBD patients and to evaluate the efficacy of therapy. Various endoscopic scores (e.g. Mayo [3], Matts [4] and Lichtiger score [5] in ulcerative colitis; CDEIS [6] or SES-CD [7] in CD) are used in clinical practice and clinical studies to assess the mucosal status in IBD patients. Since routine surveillance colonoscopy in asymptomatic IBD patients without dysplastic lesions depending on the severity and type of IBD are recommended only every 2–15 years, the mucosal status after initiation or maintenance of a new therapy often remains unclear in most of these patients. Moreover, willingness for control colonoscopy in asymptomatic patients is low.

There is growing evidence, that mucosal healing (MH) is associated with a better long-term outcome, lower need for surgeries and hospitalisation and improved quality of life in IBD patients [8], [9], [10]. Moreover, in a statement of the European Crohn's and Colitis Organization (ECCO) regarding the impact of MH on the course of IBD, the need for further studies was addressed [11].

Therefore, we aimed to analyze in this study the real-life prevalence and predictive factors of mucosal healing in IBD patients treated with anti-TNF-alpha antibodies in a large single center cohort.

## Materials and Methods

### Ethics statement

This was a retrospective study using medical records, and statistical analysis was anonymized. The ethics committee of the University of Munich was consulted (UE number 055-13) and a formal written waiver for the need of ethics approval was obtained. Written informed consent of the patients was not obtained, since patient records and relevant data were anonymized and de-identified prior to analysis.

### Study cohorts

**Figure 1:**
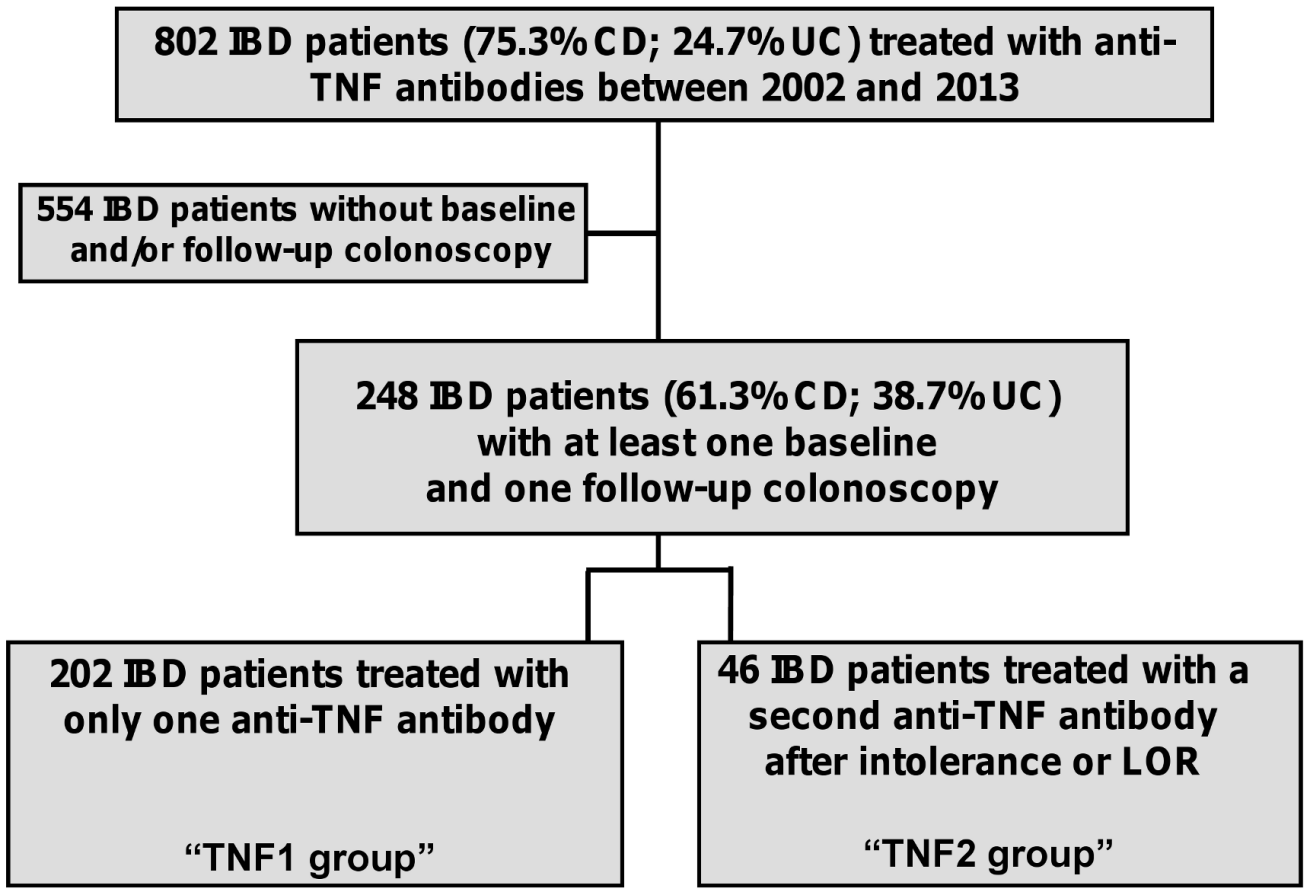
Study design. Data of 802 patients were available for analysis (75.3% patients with Crohn's disease and 24.7% patients with ulcerative colitis). In 554 IBD patients, there was no baseline and/or follow-up colonoscopy available. Accordingly, 248 patients were included in the analysis, while 202 patients were treated with one anti-TNF antibody between baseline and follow-up colonoscopy (TNF1 group) and 46 patients were treated with a second anti-TNF antibody after loss of response or intolerance to a first anti-TNF antibody (TNF2 group).

All patients, who received anti-TNF-alpha antibody treatment (infliximab or adalimumab) at our IBD center for the first time and during the time period from 2002 to 2013, were eligible for this study. Out of this cohort, patients with at least one colonoscopy before start of (baseline colonoscopy) and one during anti-TNF-alpha antibody treatment (follow-up colonoscopy) were included in this study (Figure 1). If more than one baseline or follow-up colonoscopy was available for analysis, the last colonoscopy before start of anti-TNF-alpha antibody treatment and the first colonoscopy after start of anti-TNF-alpha antibody treatment were used for analysis, respectively. Patients, who were treated with a second anti-TNF-alpha antibody after loss of response or intolerance to the first anti-TNF-alpha antibody between the time periods from baseline to follow-up colonoscopy, were assigned to the cohort “TNF2 group”. All other patients were assigned to the cohort “TNF1 group”. For further analyses, patients were divided according their disease entities (CD group and UC group).

Medical records were analyzed regarding the following parameters: demographics, C-reactive protein (CRP) value at baseline and follow-up colonoscopy, white blood cell count (WBC) at baseline and follow-up colonoscopy, age at first diagnosis, disease duration, type of anti-TNF-alpha antibody, concomitant medication, time to first anti-TNF-alpha antibody treatment, duration of anti-TNF-alpha antibody treatment, time between baseline and follow-up colonoscopy, smoking, BMI, extraintestinal manifestation, family history of IBD, reason for second (follow-up) endoscopy and hospitalization or surgery from baseline colonoscopy until last follow-up.

### Medical treatment

Adalimumab was injected at doses of 160 mg loading dose, following 80 mg and then 40 mg subcutaneously every other week. Infliximab infusions were administered using a dose of 5 mg/kg body weight intravenously over 2 hours at weeks 0, 2, 6 (induction therapy) and thereafter every 8 weeks (maintenance therapy). Thiopurines were given at the full dose (mercaptopurine 1.0–1.5 mg/kg body weight, azathioprine 2.0–2.5 mg/kg body weight) from the beginning of therapy.

### Endoscopy and histopathological analysis

Colonoscopy reports of baseline and follow-up colonoscopies were analyzed by two independent medical reviewers. The Mayo endoscopy score [3] was used for patients with UC and the SES-CD score [7] for patients with CD. Patients with macroscopic absence of IBD-defining markers and a Mayo endoscopy subscore of 0 (UC) or a SES-CD score of 0 (CD) were defined as patients with MH. All other patients were defined as patients without MH.

### Statistical analysis

Comparison of demographic and clinical characteristics of the study populations was performed using the χ^2^-test or Fisher's exact test for categorical variables and by using the Wilcoxon-Mann-Whitney test for continuous variables. Survival analyses techniques were used to model the time to surgery. All statistical analyses were performed using R (R.2.13.2). Hypotheses were tested at 5% level of significance (two-sided).

## Results

### Patients' characteristics

Data of 802 IBD patients were available for analysis (Figure 1). Out of these patients, 248 patients met the inclusion criteria and were eligible for the study. 152 patients (61.3%) had CD and 96 (38.7%) had UC.

In the TNF1 group (n = 202), 120 patients (59.4%) had CD and 82 patients (40.6%) had UC. One hundred and eight patients were female (52.5%); median of overall follow-up time for all patients was 63 months (range 3–127 months).

The TNF2 group consisted of 46 patients, 32 (69.6%) with CD and 14 (30.4%) with UC. Twenty patients were female (43.5%), median of follow-up time for the patients of the TNF2 group was 64.5 months (range 9–126). The demographic and clinical characteristics of the study cohorts are provided in table 1 and tables S1 and S6 (UC/CD subcohorts).

**Table 1:** Demographic and clinical characteristics of the study cohorts (n = 248).

	TNF1 group	TNF2 group
**Patients** (n = )	202	46
**Median age** (yrs) [Range]	38 [18–72]	43.5 [17–73]
**Median age at diagnosis** (yrs) [Range]	25 [6–63]	26 [7–68]
**Median disease duration** (yrs) [Range]	10 [0–45]	9.5 [2–44]
**Female sex** (%)	106 (52.5)	20 (43.5)
**Smoker** (%)	73 (36.2)	14 (30.4)
**Family history of IBD** (%)	27 (13.4)	2 (4.3)
**Extraintestinal manifestation** (%)	80 (39.6)	24 (52.2)
**Crohn's disease** (%)	120 (59.4)	32 (69.6)
**Ulcerative colitis** (%)	82 (40.6)	14 (30.4)
**Mean CRP-value at baseline colonoscopy** (mg/dL) [Range]	2.409 [0.1–33.8]	2.425 [0.1–15.4]
**Mean CRP-value at follow-up colonoscopy** (mg/dL) [Range]	1.341 [0.1–23.1]	1.351 [0.1–10.1]
**Mean WBC at baseline colonoscopy** (G/L) [Range]	9.263 [2.3–23.1]	9.697 [4.4;23.9]
**Mean WBC at follow-up colonoscopy (G/L)** [Range]	7.493 [1.6;17.3]	8.389 [2.7;19.5]
**Thiopurine treatment ever** (%)	172 (85.1)	44 (95.7)
**Median thiopurine treatment duration** (months) [Range]	40 [0;211]	60.5 [43;237]
**Infliximab treated patients** (%)	188 (93.1)	4 (8.7)
**Adalimumab treated patients** (%)	14 (6.9)	42 (91.3)
**Anti-TNF-alpha antibody and thiopurine treated patients at follow-up** (%)	39 (19.3)	5 (10.9)
**Median duration infliximab treatment** (months) [Range]	11 [0–70]	18 [1–39]
**Median duration adalimumab treatment** (months) [Range]	6 [1–41]	10.5 [0–44]
**Median time to first anti-TNF-alpha antibody treatment** (years) [Range]	7 [0–42]	7 [0–39]
**Median time from baseline to follow-up colonoscopy** (months) [Range]	19 [0–123]	39.5 [1–104]
**Median time from first to second anti-TNF-alpha antibody treatment** (months) [Range]	n/a	7.5 [0–68]
**Patients with surgery until follow-up** (%)	34 (16.8)	10 (23.8)
**Patients hospitalized until follow-up** (%)	57 (28.2)	18 (39.1)
**Median follow-up** (months) [Range]	63 [3–127]	64.5 [9–126]

In the TNF2 group, 42 patients were switched from infliximab to adalimumab and 4 patients were switched from adalimumab to infliximab. Reasons for the switch from a first anti-TNF-alpha antibody to a second anti-TNF-alpha antibody in these patients were allergic reactions (n = 30; 65.2%), intolerance (n = 3; 6.5%) and loss of response (n = 13; 28.3%).

The most common reason for the second (follow-up) endoscopy was routine control colonoscopy (174 patients = 86.1% in the TNF1 group, and 40 patients = 87.0% in the TNF2 group). Only a minority of the patients had second (follow-up) endoscopy due to disease flares (28 = 13.9% in the TNF1 group, and 6 patients = 13.0% in the TNF2 group).

The median time from first anti-TNF-alpha antibody treatment to follow-up colonoscopy was 11 months in the TNF1 group and 25.5 months in the TNF2 group.

### Mucosal healing at follow-up colonoscopy

**Figure 2:**
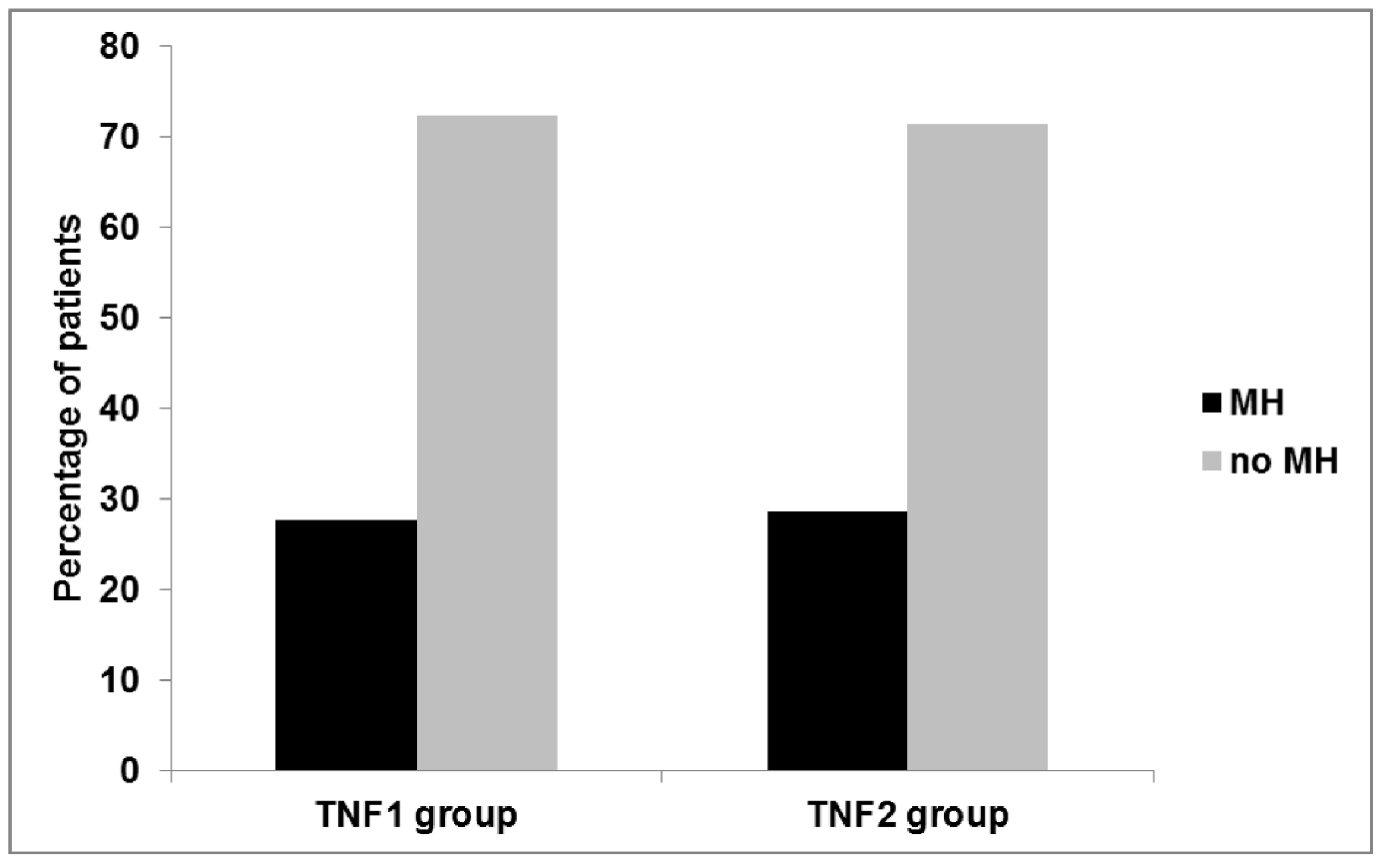
Rates of mucosal healing in both groups. In the TNF1 group, 146 patients (72.3%) had no MH, while 56 patients (27.7%) had MH. In the TNF2 group, 13 patients (28.6%) patients had MH, while 33 patients (71.4%) had no MH at follow-up colonoscopy.

In the TNF1 group, 56 patients (27.7%) had complete mucosal healing (as defined in the Methods section; Mayo endoscopy score 0 or SES-CD score 0) at follow-up colonoscopy, while 146 patients (72.3%) had no mucosal healing (Figure 2).

In the TNF2 group, 13 patients (28.3%) patients had complete mucosal healing at follow-up colonoscopy, while 33 patients (71.4%) had no mucosal healing at follow-up colonoscopy (Figure 2).

In the subanalysis of UC patients (n = 96), in the TNF1 group (n = 82) 23 patients (28.0%) patients had complete mucosal healing at follow-up colonoscopy, while 59 patients (72.0%) had no mucosal healing at follow-up colonoscopy (Table S2). In the TNF2 group (n = 14), 5 patients (35.7%) had complete mucosal healing at follow-up colonoscopy, while 9 patients (64.3%) had no mucosal healing at follow-up colonoscopy (Table S3).

In the subanalysis of CD patients (n = 152), in the TNF1 group (n = 120) 33 patients (27.5%) patients had complete mucosal healing at follow-up colonoscopy, while 87 patients (72.5%) had no mucosal healing at follow-up colonoscopy (Table S7). In the TNF2 group (n = 32), 8 patients (25.0%) patients had complete mucosal healing at follow-up colonoscopy, while 24 patients (75.0%) had no mucosal healing at follow-up colonoscopy (Table S8).

### Univariate analyses reveals serum CRP levels as predictive factor for mucosal healing at follow-up colonoscopy

In patients with MH, CRP values of follow-up colonoscopy were significantly lower compared to CRP values at baseline colonoscopy (median 0.9 mg/dL at baseline vs. 0.3 mg/dL at follow-up; p = 0.002), while there was no significant difference of CRP values from baseline to follow-up colonoscopy in patients without MH (median 1.1 mg/dL at baseline vs. 0.55 mg/dL at follow-up; p = 0.07; Table 2). Interestingly, combination therapy with thiopurines and anti-TNF-alpha antibodies had no influence on mucosal healing status (10 patients with mucosal healing vs. 29 patients without mucosal healing; p = 1.0).

**Table 2:** Demographic and clinical characteristics of the TNF1 group (n = 202) regarding MH.

	MH	No MH	p-value	OR [95%CI]
**Patients** (n = )	56 (27.7)	146 (72.3)	N/A	N/A
**Median age** (yrs) [Range]	38 [19;67]	37.5 [18;72]	0.961	1.001 [0.978;1.023]
**Median age at diagnosis** (yrs) [Range]	27.5 [14;48]	25 [6;63]	0.665	1.006 [0.980;1.033]
**Median disease duration** (yrs) [Range]	9.5 [0;33]	10 [0;45]	0.719	0.994 [0.961;1.027]
**Female sex** (%)	31 (55.4)	75 (51.4)	0.644	0.864 [0.465;1.605]
**Smoker** (%)	24 (42.9)	49 (33.6)	0.361	0.875 [0.658;1.165]
**Family history of IBD** (%)	3 (5.4)	24 (16.4)	0.048	3.556 [1.012;12.493]
**Extraintestinal manifestation** (%)	23 (41.1)	57 (39)	0.872	0.872 [0.428;1.778]
**Diagnosis Crohn's disease/Ulcerative colitis**	33/23	87/59	0.932	0.973 [0.520;1.821]
**Mean (median) CRP-value at baseline colonoscopy** (mg/dL) [Range]	2.441 (0.9) [0.1;14.8]	2.398 (1.1) [0.1;33.8]	0.948	0.997 [0.910;1.093]
**Mean (median) CRP-value at follow-up colonoscopy** (mg/dL) [Range]	0.583 (0.3) [0.1;4.3]	1.641 (0.550) [0.1;23.1]	0.040	1.705 [1.180;2.464]
**Mean (median) WBC at baseline colonoscopy** (G/L) [Range]	9.234 (8.5) [2.3;19.7]	9.274 (8.5) [3.5;23.1]	0.951	1.003 [0.916;1.098]
**Mean (median) WBC at follow-up colonoscopy (G/L)** [Range]	6.531(6.3) [1.6;14.6]	7.882 (7.700) [3.3;17.3]	0.02	1.250 [1.084;1.442]
**Thiopurine treatment ever** (%)	46 (82.1)	126 (86.3)	0.906	0.906 [0.176;4.649]
**Median thiopurine treatment duration** (months) [Range]	24 [4;104]	44.5 [0;211]	0.261	1.013 [0.990;1.037]
**Infliximab treated patients** (%)	53 (94.6)	135 (92.5)	0.7615	N/A
**Adalimumab treated patients** (%)	3 (5.4)	11 (7.5)	0.7615	N/A
**Anti-TNF-alpha antibody and thiopurine treated patients at follow-up** (%)	10 (17.9)	29 (19.9)	1.000	N/A
**Median duration infliximab treatment** (months) [Range]	12.5 [0;69]	10 [0;70]	0.4191	0.992 [0.971;1.012]
**Median duration adalimumab treatment** (months) [Range]	12 [0;69]	10 [0;70]	0.727	0.996 [0.975;1.018]
**Median thiopurine treatment duration** (months) [Range]	22.5 [1;41]	6 [1]; [25]	0.078	0.883 [0.769;1.014]
**Median time to first anti-TNF-alpha antibody treatment** (years) [Range]	6 [0;30]	7 [0;42]	0.835	0.996 [0.960;1.033]
**Median time from baseline to follow-up colonoscopy** (months) [Range]	19.5 [0;106]	19 [0;123]	0.853	0.999 [0.984;1.013]
**Patients with surgery until follow-up** (%)	3 (5.4)	31 (21.2)	0.006	N/A
**Patients hospitalized until follow-up** (%)	10 (17.9)	47 (32.2)	0.054	N/A
**Median follow-up** (months) [Range]	70 [22;127]	60 [3;125]	0.141	0.991 [0.980;1.003]

Similar to the TNF1 group, in the TNF2 group CRP values at follow-up colonoscopy were lower in patients with mucosal healing compared to baseline values, but not statistically significant (CRP median 0.6 mg/dL at baseline vs. 0.3 mg/dL at follow-up; p = 0.16). In patients without MH, CRP values at follow-up were not significantly different to CRP values at baseline colonoscopy (CRP median 0.7 mg/dL at baseline vs. 0.8 mg/dL at follow-up; p = 0.14, Table 3).

**Table 3:** Demographic and clinical characteristics of the TNF2 group (n = 46) regarding MH.

	MH	No MH	p-value	OR [95%CI]
**Patients** (n = )	13 (28.6)	33 (71.4)	N/A	N/A
**Median age** (yrs) [Range]	44 [26;73]	42 [17;69]	0.277	0.974 [0.929;1.021]
**Median age at diagnosis** (yrs) [Range]	33 [7;68]	25 [10;55]	0.142	0.965 [0.921;1.012]
**Median disease duration** (yrs) [Range]	8 [3;39]	12 [2;44]	0.607	1.018 [0.950;1.091]
**Female sex** (%)	7 (53.8)	13 (39.4)	0.376	0.557 [0.153;2.034]
**Smoker** (%)	4 (30.8)	10 (30.3)	0.530	0.763 [0.327;1.777]
**Family history of IBD** (%)	1 (7.7)	1 (3.0)	0.477	0.355 [0.02;6.172]
**Extraintestinal manifestation** (%)	3 (23.1)	21 (63.6)	0.035	5.091 [1.12;23.142]
**Diagnosis Crohn's disease/Ulcerative colitis**	8/5	24/9	0.460	0.60 [0.155;2.325]
**Mean (median) CRP-value at baseline colonoscopy** (mg/dL) [Range]	1.167 (0.6) [0.1;4.9]	2.902 (0.67) [0.1;15.4]	0.212	1.329 [0.850;2.079]
**Mean (median) CRP-value at follow-up colonoscopy** (mg/dL) [Range]	0.380 (0.300) [0.1;0.8]	1.645 (0.8) [0.1;10.1]	0.003	0.31 [0.144;0.661]
**Mean (median) WBC at baseline colonoscopy** (G/L) [Range]	9.144 (8.1) [6.8;12.6]	10.064 (9.1) [4.4;23.9]	0.581	1.075 [0.831;1.390]
**Mean (median) WBC at follow-up colonoscopy (G/L)** [Range]	8.265 (7.0) [4;19.3]	8.430 (7.70) [2.7;19.5]	0.904	1.011 [0.849;1.204]
**Thiopurine treatment ever** (%)	13 (100)	31 (93.9)	1.000	N/A
**Median thiopurine treatment duration** (months) [Range]	75 [75;75]	46 [43;237]	0.725	1.006 [0.971;1.043]
**Infliximab after adalimumab treated patients (%)**	1 (7.7)	3 (9.1)	1.000	N/A
**Adalimumab after infliximab treated patients (%)**	12 (92.3)	30 (91.9)	1.000	N/A
**Anti-TNF-alpha antibody and thiopurine treated patients at follow-up** (%)	1 (7.7)	4 (12.1)	1.000	N/A
**Median duration infliximab treatment** (months) [Range]	17 [9;95]	29 [0;68]	0.985	1.000 [0.969;1.032]
**Median duration adalimumab treatment** (months) [Range]	17 [17;17]	19 [1;39]	0.865	1.015 [0.855;1.205]
**Median thiopurine treatment duration** (months) [Range]	9.5 [2;44]	13 [0;42]	0.458	0.981 [0.931;1.033]
**Median time to first anti-TNF-alpha antibody treatment** (years) [Range]	5 [0;37]	8 [0;39]	0.625	1.017 [0.950;1.089]
**Median time from baseline to follow-up colonoscopy** (months) [Range]	45 [14;90]	33 [1;104]	0.332	0.988 [0.964;1.012]
**Median time from first to second anti-TNF-alpha antibody treatment (months)** [Range]	5 [1;68]	10 [0;56]	0.576	1.013 [0.968;1.06]
**Patients with surgery until follow-up** (%)	0 (0)	10 (30.3)	0.042	N/A
**Patients hospitalized until follow-up** (%)	2 (15.4)	16 (48.5)	0.049	N/A
**Median follow-up** (months) [Range]	77 [14;123]	58 [9;126]	0.461	0.993 [0.973;1.012]

**Figure 3:**
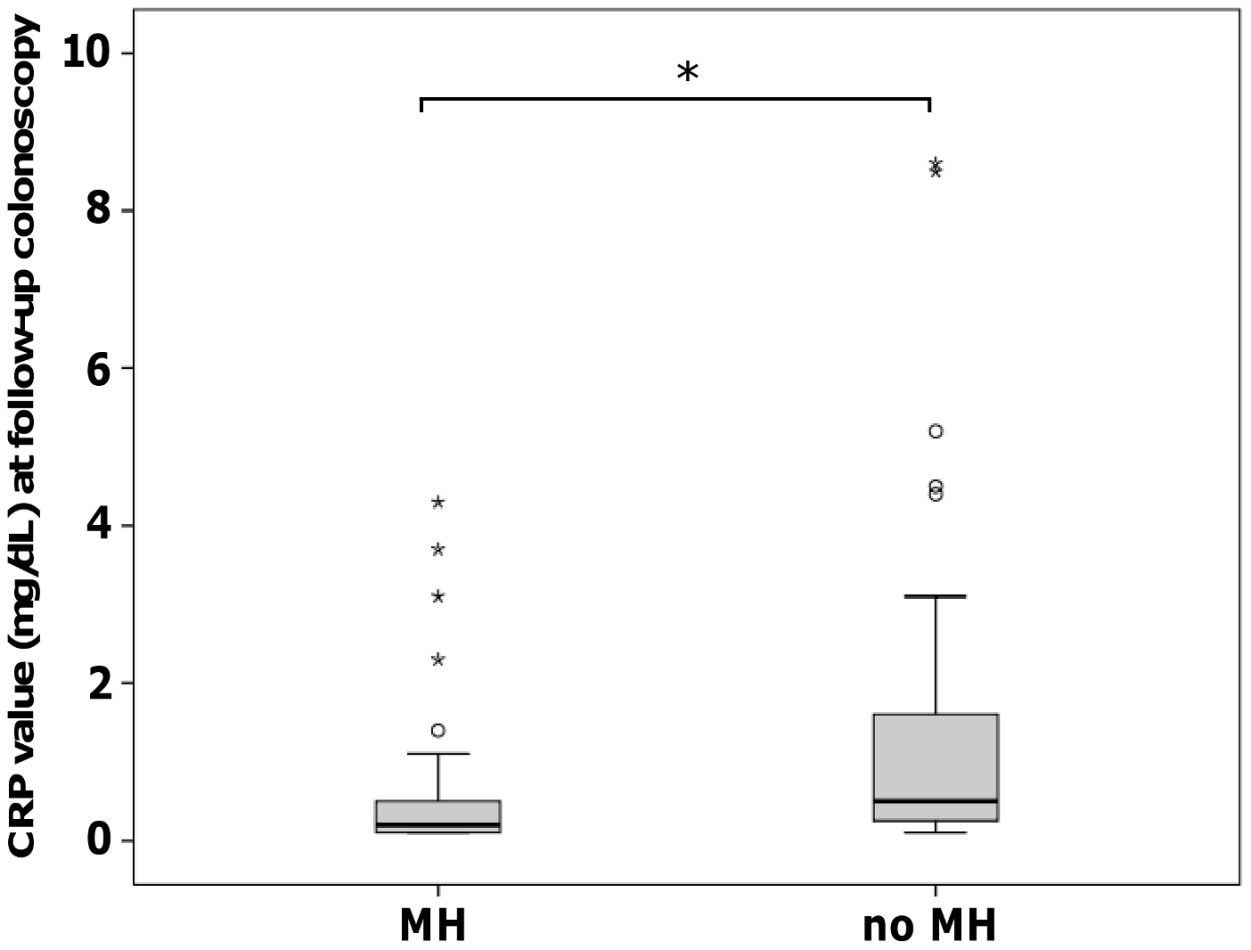
CRP values at follow-up colonoscopy (TNF1 group). At follow-up colonoscopy, CRP values were significantly lower in patients with MH compared to patients without MH (*p = 8.34E-05).

**Figure 4:**
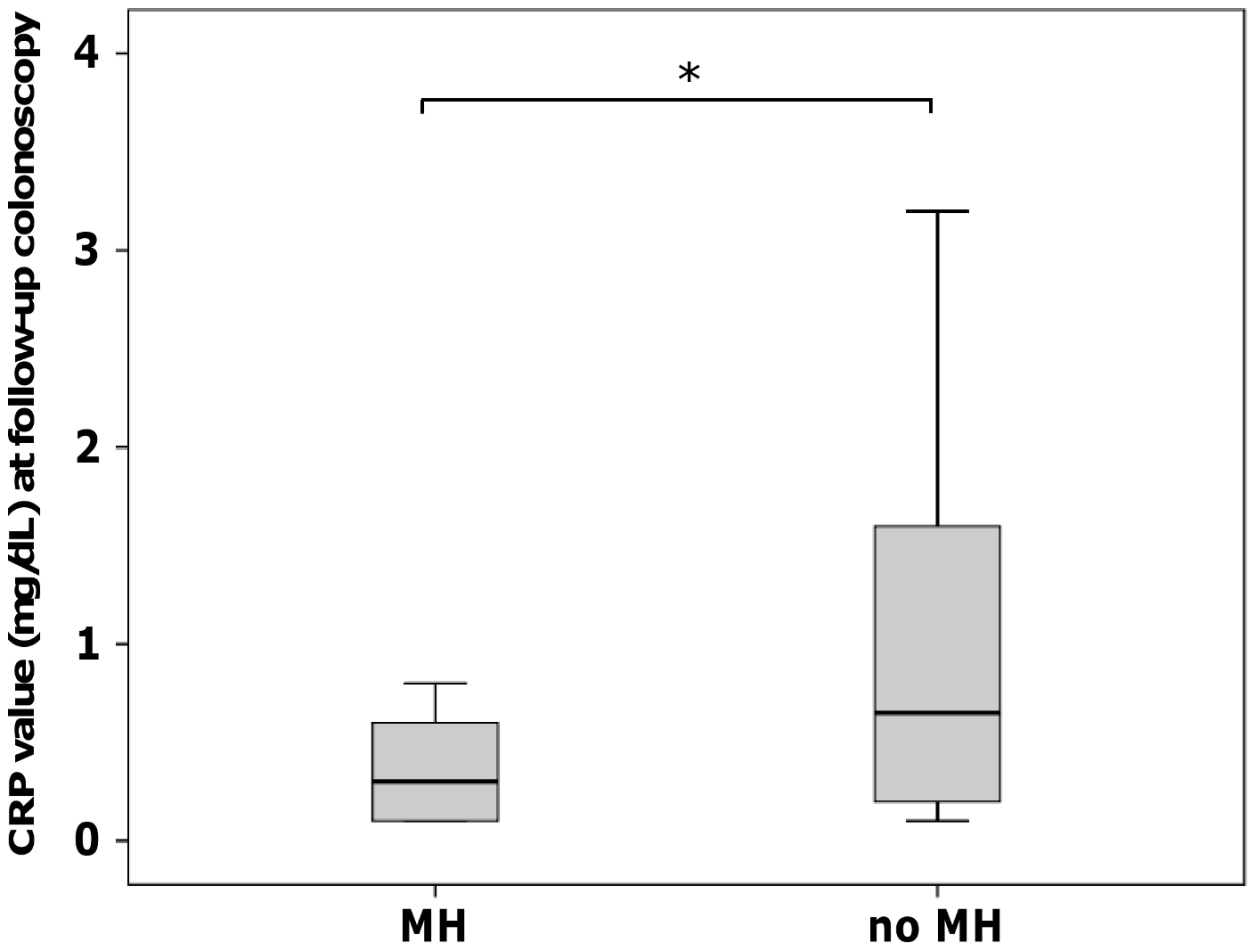
CRP values at follow-up colonoscopy (TNF2 group). At follow-up colonoscopy, there was a trend for lower CRP in patients with MH compared to patients without MH (*p = 0.002).

At baseline colonoscopy, CRP values were not significantly different in patients with or without MH in the TNF1 group (p = 0.95). However, CRP values were significantly lower at follow-up colonoscopy in patients with MH compared to patients without MH in the TNF1 group (p = 8.35E-05; Figure 3). Similar to the TNF1 group, baseline colonoscopy CRP values were not significantly different in patients with or without MH in the TNF2 group (p = 0.06). In patients with MH of the TNF2 group, CRP values at follow-up colonoscopy were significantly lower compared to patients without MH (p = 0.002; Figure 4).

In the subanalysis of UC patients, CRP values in the TNF1 group at follow-up colonoscopy were significantly lower in patients with MH compared to patients without MH (CRP median 0.10 mg/dL (MH) vs. 0.50 mg/dL (no MH); p = 0.0002; Figure S1), while CRP values at baseline colonoscopy were not significantly different (CRP median 0.6 mg/dL (MH) vs. 1.0 mg/dL (no MH); p = 0.58). In patients with MH, CRP values of follow-up colonoscopy were significantly lower compared to CRP values at baseline colonoscopy (CRP median 0.6 mg/dL at baseline vs. 0.1 mg/dL at follow-up; p = 0.03), while there was no significant difference of CRP values from baseline to follow-up colonoscopy in patients without MH (CRP median 1.0 mg/dL at baseline vs. 0.5 mg/dL at follow-up; p = 0.10). In the TNF2 group, CRP values at follow-up colonoscopy were significantly lower in patients with mucosal healing compared to patients without MH (CRP median 0.20 mg/dL (MH) vs. 0.80 mg/dL (no MH); p = 0.03; Figure S2), while CRP values at baseline colonoscopy were not significantly different (CRP median 0.55 mg/dL (MH) vs. 0.6 mg/dL (no MH); p = 0.26). In patients with MH, CRP values of follow-up colonoscopy were significantly lower compared to CRP values at baseline colonoscopy (CRP median 0.55 mg/dL at baseline vs. 0.20 mg/dL at follow-up; p = 0.01); there was no significant difference of CRP values from baseline to follow-up colonoscopy in patients without MH (CRP median 0.6 mg/dL at baseline vs. 0.8 mg/dL at follow-up; p = 0.80).

In the subanalysis of CD patients, CRP values in the TNF1 group at follow-up colonoscopy were significantly lower in patients with MH compared to patients without MH (CRP median 0.40 mg/dL (MH) vs. 0.60 mg/dL (no MH); p = 0.01; Figure S3), while CRP values at baseline colonoscopy were not significantly different (CRP median 1.5 mg/dL (MH) vs. 1.15 mg/dL (no MH); p = 0.57). In patients with MH, CRP values of follow-up colonoscopy were significantly lower compared to CRP values at baseline colonoscopy (median 1.5 mg/dL at baseline vs. 0.4 mg/dL at follow-up; p = 0.02), while there was no significant difference of CRP values from baseline to follow-up colonoscopy in patients without MH (median 1.15 mg/dL at baseline vs. 0.6 mg/dL at follow-up; p = 0.23). In the TNF2 group, CRP values at follow-up colonoscopy were significantly lower in patients with MH compared to patients without MH (CRP median 0.60 mg/dL (MH) vs. 0.70 mg/dL (no MH); p = 0.01; Figure S4), while CRP values at baseline colonoscopy were not significantly different (CRP median 0.6 mg/dL (MH) vs. 2.3 mg/dL (no MH); p = 0.08). However, in patients with MH, CRP values of follow-up colonoscopy were not significantly lower compared to CRP values at baseline colonoscopy (median 0.6 mg/dL at baseline vs. 0.6 mg/dL at follow-up; p = 0.24); there was also no significant difference of CRP values from baseline to follow-up colonoscopy in patients without MH (median 2.3 mg/dL at baseline vs. 0.7 mg/dL at follow-up; p = 0.16).

### Mulitvariate analyses reveals serum CRP levels as predictive factor for mucosal healing at follow-up colonoscopy

Multivariate analysis confirmed CRP at follow-up colonoscopy as predictive factor for MH (p = 0.01) in the TNF1 group, while CRP at follow-up colonoscopy was not predictive for MH in the TNF2 group (p = 0.11, Tables 4 and 5).

**Table 4:** Multivariate analysis for outcome MH in the TNF1 group (n = 202).

	p-value	OR [95%CI]
**CRP-value at baseline colonoscopy**	0.398	1.080 [0.904;1.290]
**CRP-value at follow-up colonoscopy**	0.012	1.687 [1.124;2.533]
**WBC at baseline colonoscopy**	0.685	0.976 [0.870;1.096]
**WBC at follow-up colonoscopy**	0.026	1.187 [1.021;1.380]
**Age at diagnosis**	0.869	0.901 [0.262;3.098]
**Age**	0.854	1.123 [0.326;3.865]
**Gender**	0.174	0.602 [0.289;1.251]
**Smoker**	0.428	0.871 [0.620;1.225]
**Duration anti-TNF-alpha antibody treatment**	0.691	0.992 [0.951;1.034]
**Time to first anti-TNF-alpha antibody treatment**	0.102	1.176 [0.968;1.429]
**Time from baseline to follow-up colonoscopy**	0.893	1.002 [0.967;1.039]

**Table 5:** Multivariate analysis for outcome MH in the TNF2 group (n = 46).

	p-value	OR [95%CI]
**CRP-value at baseline colonoscopy**	0.197	1.976 [0.702;5.561]
**CRP-value at follow-up colonoscopy**	0.106	3.640 [0.761;17.415]
**WBC at baseline colonoscopy**	0.350	1.149 [0.859;1.538]
**WBC at follow-up colonoscopy**	0.719	0.957 [0.753;1.252]
**Age at diagnosis**	0.556	0.825 [0.436;1.563]
**Age**	0.527	1.228 [0.650;2.321]
**Gender**	0.201	0.137 [0.007;2.877]
**Smoker**	0.135	0.419 [0.134;1.312]
**Duration anti-TNF-alpha antibody treatment**	0.857	0.993 [0.924;1.068]
**Time to first anti-TNF-alpha antibody treatment**	0.231	0.600 [0.259;1.386]
**Time from baseline to follow-up colonoscopy**	0.201	1.098 [0.951;1.267]
**Time from first to second anti-TNF-alpha antibody treatment**	0.639	0.973 [0.866;1.092]

In the subanalysis of UC patients, CRP at follow-up colonoscopy was not predictive for MH in the TNF1 group (p = 0.06) and the TNF2 group (p = 0.39) (Tables S4 and S5). In the subanalysis of CD patients, CRP at follow-up colonoscopy was also not predictive for MH in the TNF1 group (p = 0.08) and the TNF2 group (p = 0.28) (Tables S9 and S10).

### Rate of hospitalization and surgery

Ten patients with MH and 47 patients without MH in the TNF1 group (p = 0.054) and 2 patients with MH and 16 patients without MH in the TNF2 group (p = 0.05) were hospitalized due to IBD (Tables 2 and 3).

**Figure 5:**
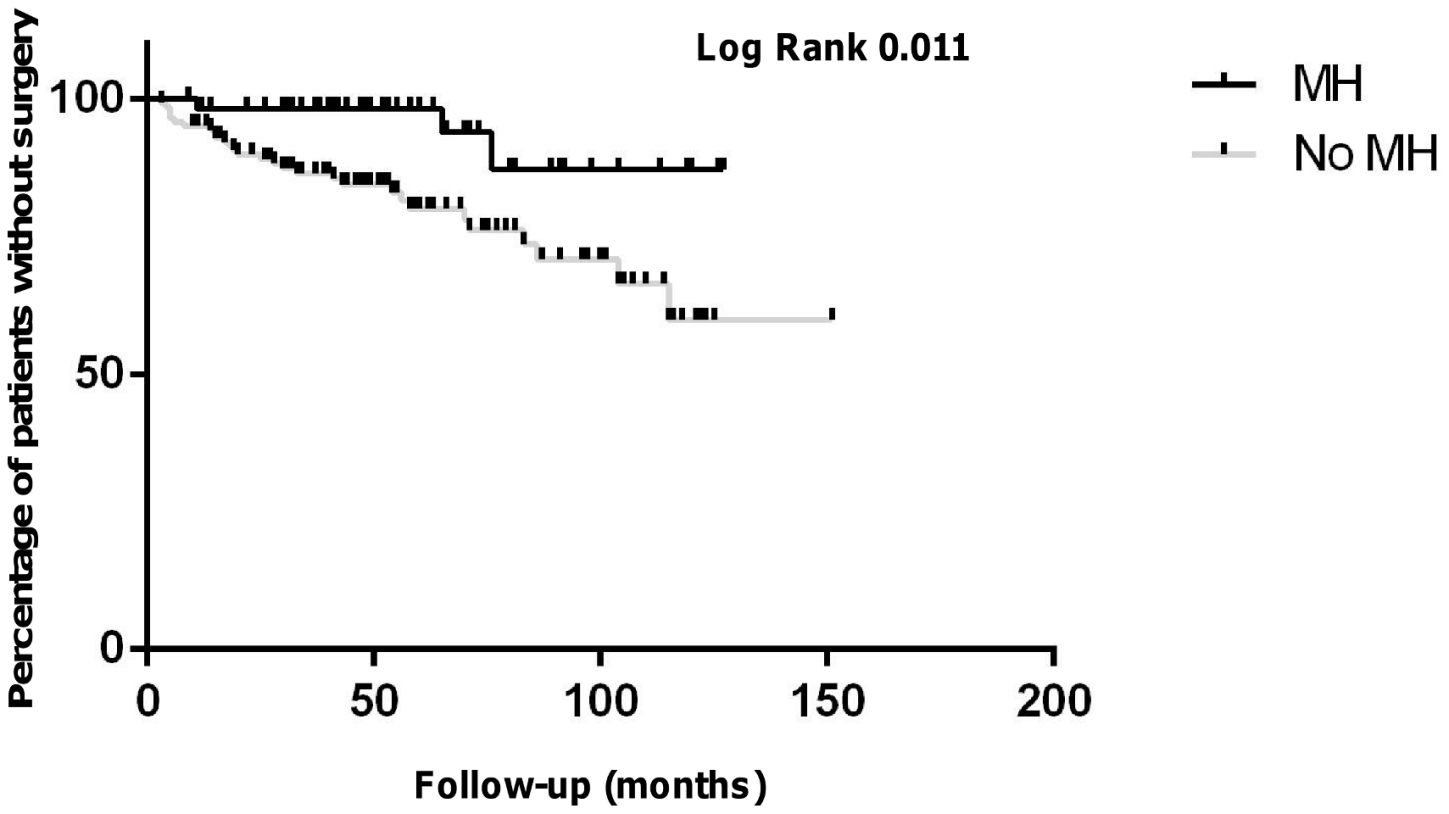
Kaplan-Mayer estimate for surgery-free time intervals in the TNF1 group over the follow-up time. During the follow-up time, 3 patients with MH underwent surgery as compared to 31 patients without MH patients (logrank p = 0.01).

**Figure 6:**
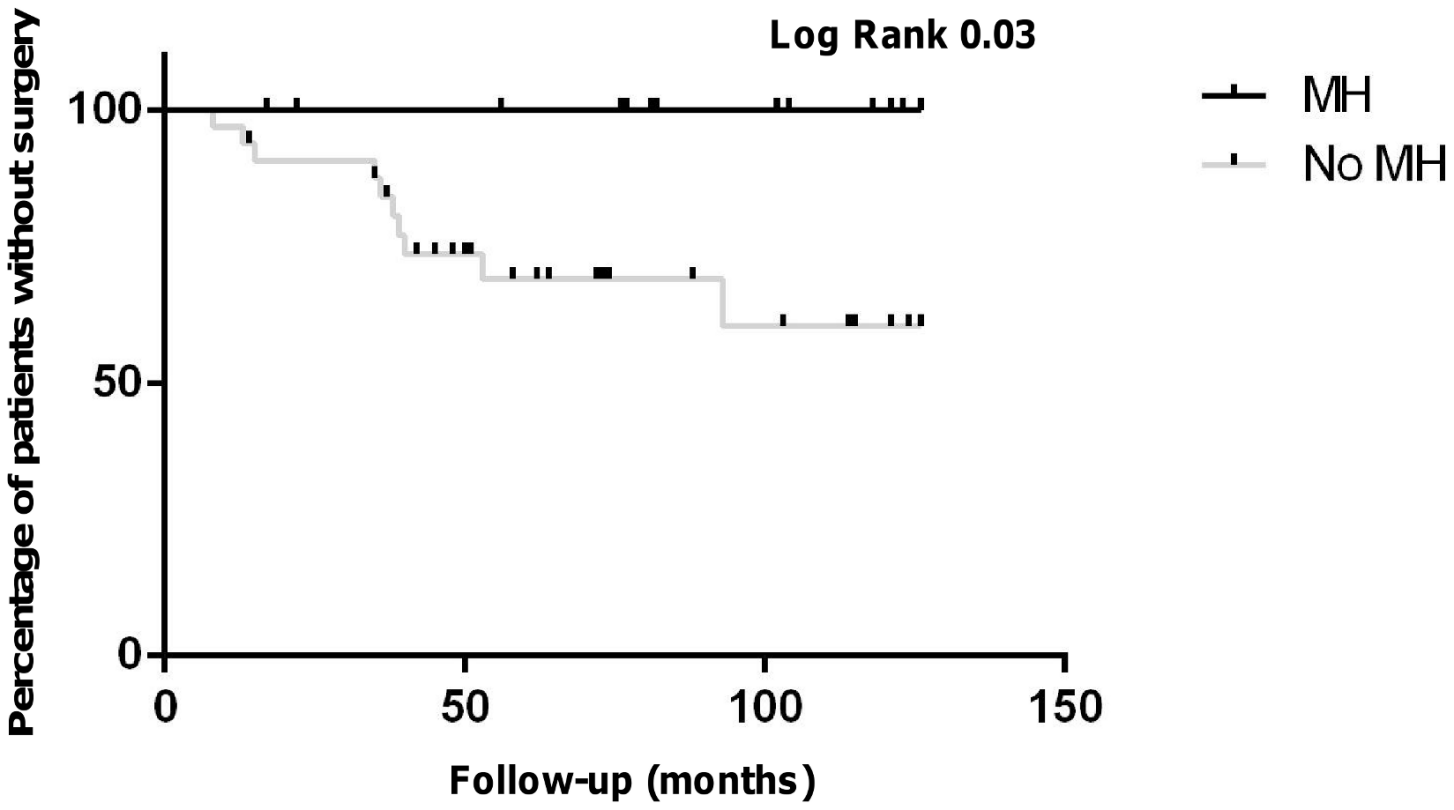
Kaplan-Mayer estimate for surgery-free time intervals in the TNF2 group over the follow-up time. During the follow-up time, no patient with MH underwent surgery as compared to 10 patients without MH (logrank p = 0.03).

Overall, 3 patients with MH and 31 patients without MH in the TNF1 group (p = 0.006) and no patient with MH and 10 patients without MH (p = 0.04) had surgery until follow-up in the TNF2 group (Tables 2 and 3). Accordingly, Kaplan-Mayer estimation revealed significantly lower need for surgery in patients with MH compared to patients without MH in both groups (Figures 5 and 6).

In the subanalysis of UC patients, 1 patients with MH and 21 patients without MH in the TNF1 group (p = 0.005) and no patient with MH and 5 patients without MH in the TNF2 group (p = 0.08) were hospitalized due to IBD. Overall, no patient with MH and 7 patients without MH in the TNF1 group (p = 0.18) and no patient with MH and 3 patients without MH (p = 0.25) had surgery until follow-up in the TNF2 group (Tables S2 and S3).

In the subanalysis of CD patients, 9 patients with MH and 26 patients without MH in the TNF1 group (p = 0.82) and 2 patients with MH and 11 patients without MH in the TNF2 group (p = 0.42) were hospitalized due to IBD. Overall, 3 patients with MH and 24 patients without MH in the TNF1 group (p = 0.049) and no patient with MH and 7 patients without MH (p = 0.14) had surgery until follow-up in the TNF2 group (Tables S7 and S8).

Kaplan-Mayer estimation revealed significantly lower need for surgery in patients with MH compared to patients without MH only in the TNF1 group of CD patients (Figures S5, S6, S7, S8).

## Discussion

Mucosal healing (MH) in patients with IBD is an important treatment goal, leading to better long-term remission rates, better quality of life, lower need for hospitalisation and surgeries, and lower rates of colorectal cancer [9], [12], [13], [14]. MH can be achieved with various treatment strategies including immunosuppressive therapies such as methotrexate or thiopurines and anti-TNF-alpha antibodies [8], [15], [16], [17], [18]. Former studies have demonstrated that corticosteroids are not suitable for maintenance of mucosal healing [19], and combination therapy with thiopurines and anti-TNF-alpha antibodies is superior compared to thiopurine monotherapy in CD regarding remission rates and MH [20]. Emerging data indicate that early use of anti-TNF-alpha antibodies lead to better long-term outcome in IBD patients by preventing mucosal damage [8], [10], [13], [21]. However, results from prospective large-scale studies are still very limited.

In our study, we found a MH prevalence of 27.7% in patients who were treated with one anti-TNF-alpha antibody (TNF1 group) and 28.3% in a second cohort which included patients who were switched to another anti-TNF-alpha antibody (TNF2 group) due to intolerance or loss of response. The MH rates in our cohort were somewhat lower than that of previously published studies which is related to the strict definition of MH in our study which required complete endoscopic MH (Mayo endoscopic subscore 0 for UC patients or SES-CD score 0 for CD patients, respectively). Of note, our study comprises a large cohort with a long follow-up time (median overall follow-up time: TNF1 group 63 months, TNF2 group 64.5 months) compared to most other clinical studies which have much shorter follow-up times. Another new finding of our study is that switch from a first anti-TNF-alpha antibody to a second anti-TNF-alpha antibody had no significant impact on MH rates, independently from the reason for switch. However, since most of our patients were switched due to allergic reaction or intolerance, MH rates might be lower when switch is done due to loss of response. Similar to recent studies, which demonstrated a lower need for surgeries and hospitalization in patients who have MH [9], [14], we confirmed these findings. Regarding predictive factors for MH, CRP values in the TNF1 group and in the TNF2 group at follow-up colonoscopy were significantly lower in patients with MH, when compared to patients without MH. Multivariate analysis confirmed CRP as a predictive marker in the TNF1 group, while smoking was negatively associated with MH in this group.

When dividing our patient cohort regarding their disease entity (CD and UC), the MH rates remain similar to the overall cohorts (CD: TNF1 group 27.5%, TNF2 group 25.0%; UC: TNF1 group 28.0%, TNF2 group 35.7%). In univariate analyses, both in CD and UC patients, low CRP levels at follow-up were associated with MH. However, multivariate analyses could not confirm CRP at follow-up as a predictive marker in both groups. This might be explained by the relative small sample size, especially in the TNF2 groups.

Interestingly, a recent study also demonstrated normalisation of CRP as a strong predictor of efficacy and mucosal healing during the first year of adalimumab therapy in CD [18]. In this study, logistic regression analysis revealed normalisation of CRP (defined as CRP<10 mg/L) at week 12 after initiation of adalimumab therapy as a predictive factor for MH (p<0.001). Another study also showed that CRP correlated with MH (p = 0.033) in CD patients treated with infliximab [22].

The anti-TNF-alpha antibodies infliximab and adalimumab have the potential to induce and maintain MH in IBD [13], [17], [23]. Moreover, there is evidence that early infliximab-induced MH is associated with a better long-term outcome and a lower need for major abdominal surgeries in CD and UC [8], [9], [24]. A recent retrospective analysis demonstrated that infliximab induced MH in 45% of CD patients 3 months after start of inflixmab which was highly predictive for MH after 12 months [25]. One possible mechanism by which infliximab induces mucosal healing in UC is down-regulation of basic fibroblast growth factor/syndecan 1 [26]. Another study revealed a significant induction of regulatory macrophages in patients with mucosal healing after treatment with infliximab which was more pronounced in patients receiving infliximab/azathioprine combination treatment [27]. This might be an explanation for the better outcome of this combination treatment regime in clinical practice and as demonstrated in the SONIC trial [20]. However, in our study combination therapy with anti-TNF-alpha antibody and thiopurines was not associated with a higher rate of MH. Other important mechanisms by which anti-TNF-alpha antibodies may promote MH are downregulation of proinflammatory cytokines/chemokines [28], matrix-metalloproteinases, tissue inhibitors of metalloproteinases [29] and apoptosis/necroptosis [30].

One may speculate if early start of anti-TNF-alpha antibody treatment could have improved the rates of MH in our study cohort, since most of the patients in our study underwent step-up strategies and were treated with an anti-TNF-alpha antibody only 7 years (median) after the first diagnosis of IBD. In the large anti-TNF-alpha antibody landmark trials, MH rates are varying with the different time points of assessment. In CD patients, infliximab induced MH rates ranging from 29% to 45% [20], [31] and 27% with adalimumab [32]. In studies with UC patients, MH rates are higher and ranged from 47% with adalimumab [23] and 60% with infliximab [24]. However, in contrast to these anti-TNF-alpha antibody studies, in which patients have routine colonoscopy after a defined time point after initiation of anti-TNF-alpha antibody treatment, in clinical practice patients without clinical symptoms are often not willing to undergo colonoscopy. Accordingly, data from these patients are missing and in a real life situation there might be higher rates of MH in anti-TNF-alpha antibody-treated patients who have no colonoscopy after start of anti-TNF-alpha antibody therapy, since clinical activity was obviously low and there was no need for routine diagnostic colonoscopy.

There are some limitations of our study, mostly due to its retrospective nature: first, there were limited data on clinical scores like CDAI/CAI. However, there is evidence that CDAI and CAI as subjective scoring systems are not suitable to determine MH [33]. Moreover, only in a few patients neutrophil-derived fecal stool markers like calprotectin were available to be correlated with MH. Second, time periods between baseline and follow-up colonoscopy were varying (see table 1). However, there was no significant difference in the time interval between baseline and follow-up colonoscopy among patients with and without MH. In the majority of our patients (88%), the main reason for second (follow-up) colonoscopy was routine control of ongoing anti-TNF-alpha antibody therapy, and only few colonoscopies were performed due to flare of the disease (12%). Accordingly, CRP values at baseline colonoscopy were not significantly different in both groups, which suggest similar clinical disease activity.

As noted above, colonoscopy as an invasive procedure has a low acceptance rate in asymptomatic IBD patients. Other non-invasive diagnostic modalities for the assessment of MH like fecal calprotectin [34], [35], lactoferrin [35] or combination of fecal calprotectin and clinical activity scores (e.g. Harvey-Bradshaw index) [36] and radiological techniques (e.g. MR enteroclysis [37]) may serve as additional or alternative markers of MH in the near future. However, despite the presence of complete MH, histological findings which predict disease relapse like basal plasma cell infiltration in UC patients [38], are valuable markers and should be additionally taken into account when monitoring IBD patients.

In summary, in nearly 30% of IBD patients treated with anti-TNF-alpha antibodies we observed complete MH. Switch from one anti-TNF-alpha antibody to a second anti-TNF-alpha antibody had no significant impact on MH rates in our study. We confirmed former findings that the need for surgeries and hospitalisation was lower in patients with MH than in patients without MH. After initiation of an anti-TNF-alpha antibody therapy, low CRP values at follow-up colonoscopy compared to a baseline colonoscopy were associated with MH. Therefore, in clinical practice the CRP value seems to be an easy-to-use marker for MH in IBD patients. Further prospective studies comparing step-up versus early interventional top-down strategies and defined colonoscopy intervals after the start of anti-TNF-alpha antibody treatment are warranted to assess their value for complete MH and long term outcome of these patients.

## Supporting Information

Figure S1 **CRP values of UC patients at follow-up colonoscopy (TNF1 group).** At follow-up colonoscopy, CRP values were significantly lower in patients with MH compared to patients without MH (*p = 0.0002).
(TIF)

Figure S2 **CRP values of UC patients at follow-up colonoscopy (TNF2 group).** At follow-up colonoscopy, CRP values were significantly lower in patients with MH compared to patients without MH (*p = 0.03).
(TIF)

Figure S3 **CRP values of CD patients at follow-up colonoscopy (TNF1 group).** At follow-up colonoscopy, CRP values were significantly lower in patients with MH compared to patients without MH (*p = 0.01).
(TIF)

Figure S4 **CRP values of CD patients at follow-up colonoscopy (TNF2 group).** At follow-up colonoscopy, CRP values were significantly lower in patients with MH compared to patients without MH (*p = 0.01).
(TIF)

Figure S5 **Kaplan-Mayer estimate for surgery-free time intervals in UC patients (TNF1 group) during the follow-up time.** During the follow-up time, no patient with MH underwent surgery as compared to 7 patients without MH patients (logrank p = 0.09).
(TIF)

Figure S6 **Kaplan-Mayer estimate for surgery-free time intervals in UC patients (TNF2 group) during the follow-up time.** During the follow-up time, no patient with MH underwent surgery as compared to 3 patients without MH patients (logrank p = 0.17).
(TIF)

Figure S7 **Kaplan-Mayer estimate for surgery-free time intervals in CD patients (TNF1 group) during the follow-up time.** During the follow-up time, 3 patients with MH underwent surgery as compared to 24 patients without MH patients (logrank p = 0.04).
(TIF)

Figure S8 **Kaplan-Mayer estimate for surgery-free time intervals in CD patients (TNF2 group) during the follow-up time.** During the follow-up time, no patient with MH underwent surgery as compared to 7 patients without MH patients (logrank p = 0.09).
(TIF)

Table S1 **Demographic and clinical characteristics of the UC study cohort (n = 96).**
(DOC)

Table S2 **Demographic and clinical characteristics of the UC TNF1 group (n = 82) regarding MH.**
(DOC)

Table S3 **Demographic and clinical characteristics of the UC TNF2 group (n = 14) regarding MH.**
(DOC)

Table S4 **Multivariate analysis for outcome MH in the UC TNF1 group.**
(DOC)

Table S5 **Multivariate analysis for outcome MH in the UC TNF2 group.**
(DOC)

Table S6 **Demographic and clinical characteristics of the CD study cohort (n = 152).**
(DOC)

Table S7 **Demographic and clinical characteristics of the CD TNF1 group (n = 120) regarding MH.**
(DOC)

Table S8 **Demographic and clinical characteristics of the CD TNF2 group (n = 32) regarding MH.**
(DOC)

Table S9 **Multivariate analysis for outcome MH in the MC TNF1 group.**
(DOC)

Table S10 **Multivariate analysis for outcome MH in the MC TNF2 group.**
(DOC)
