# Supplementary figures and images for: Rate and Predictors of Mucosal Healing in Patients with Inflammatory Bowel Disease Treated with Anti-TNF-Alpha Antibodies
Source: PLoS One. 2014 Jun 16;9(6):e99293. doi: 10.1371/journal.pone.0099293 (PMC4059645; doi:10.1371/journal.pone.0099293)

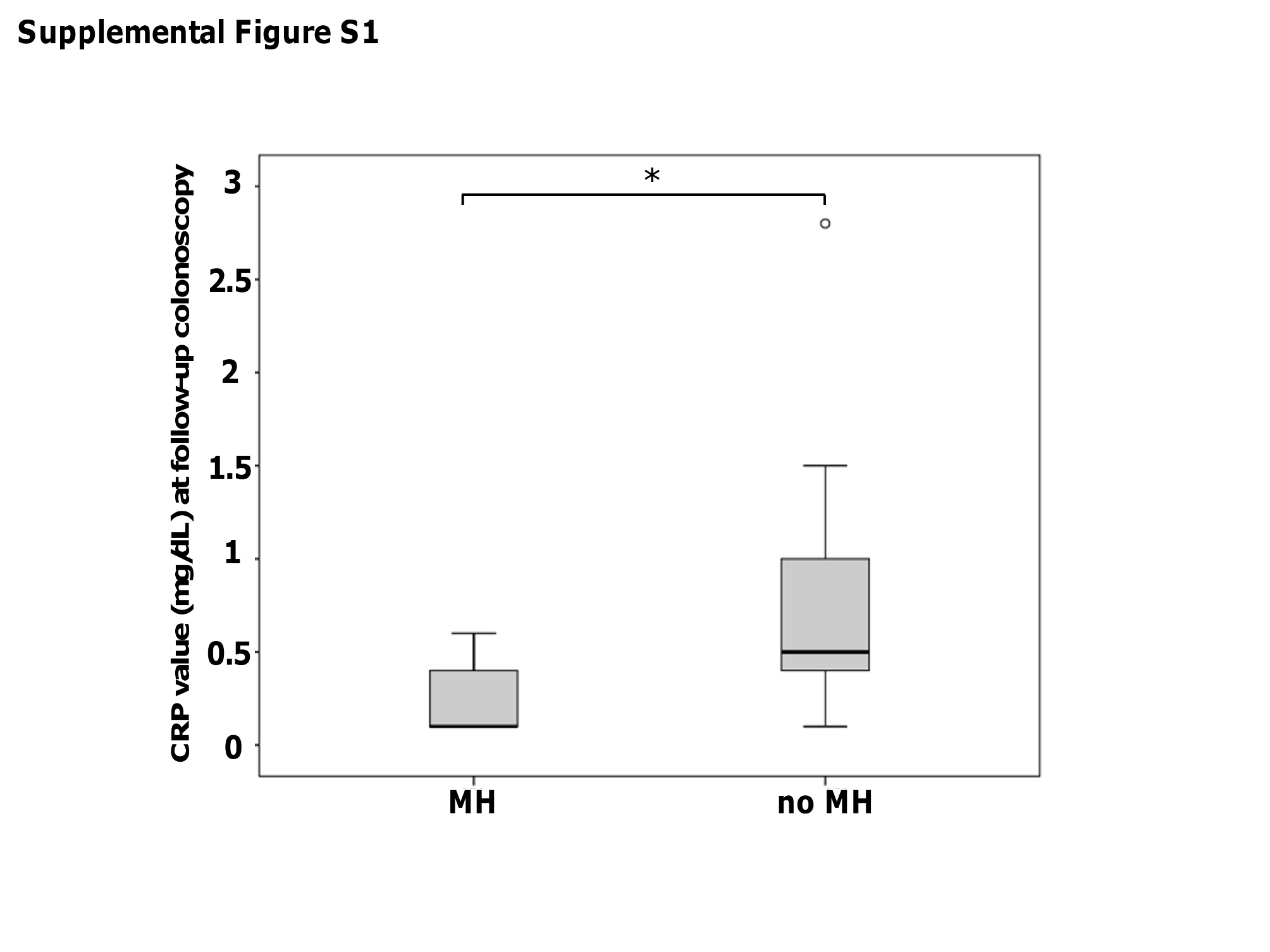

Supplement: Figure S1 — CRP values of UC patients at follow-up colonoscopy (TNF1 group). At follow-up colonoscopy, CRP values were significantly lower in patients with MH compared to patients without MH (*p = 0.0002). (TIF) [file pone.0099293.s001.tif]

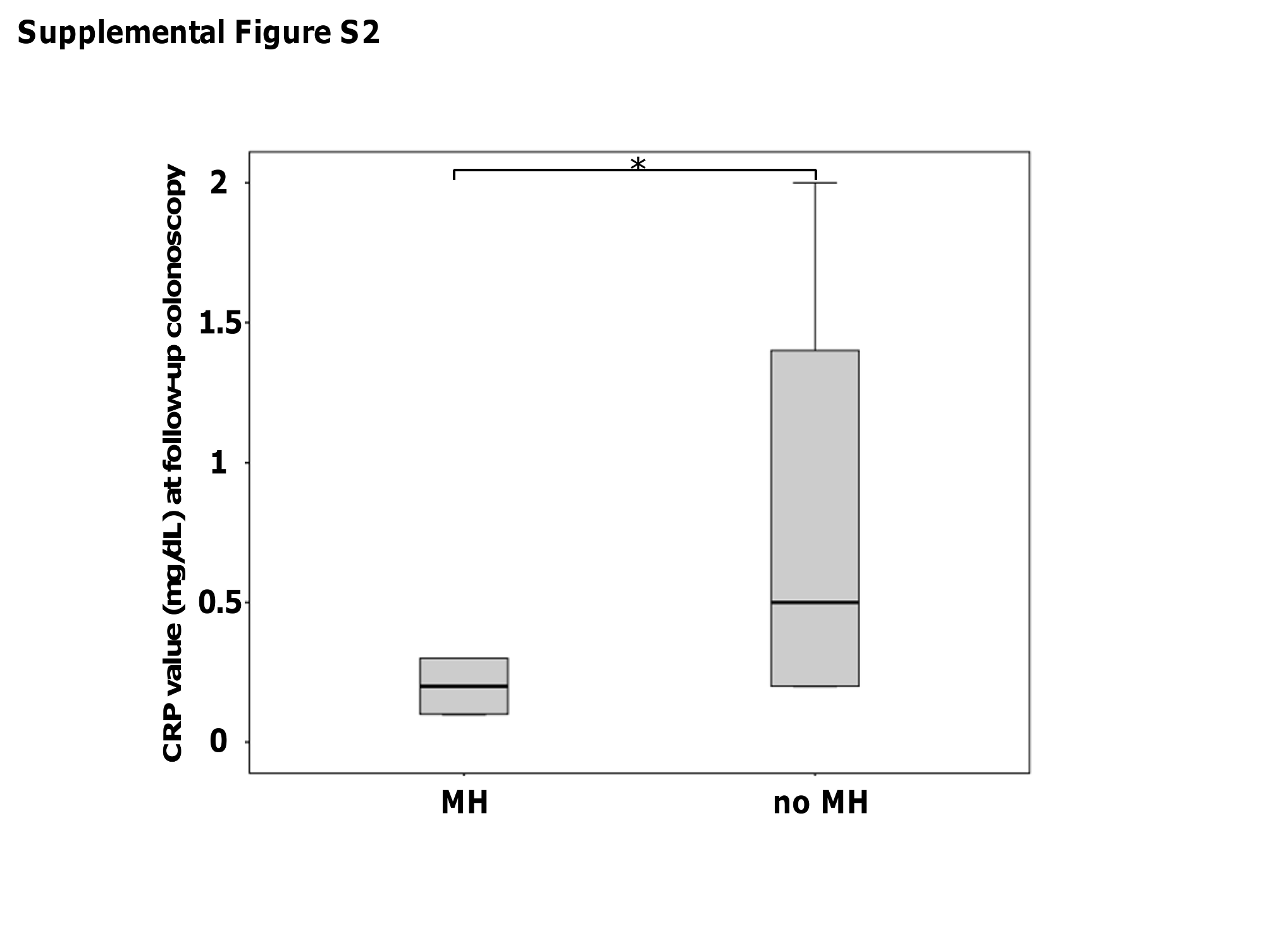

Supplement: Figure S2 — CRP values of UC patients at follow-up colonoscopy (TNF2 group). At follow-up colonoscopy, CRP values were significantly lower in patients with MH compared to patients without MH (*p = 0.03). (TIF) [file pone.0099293.s002.tif]

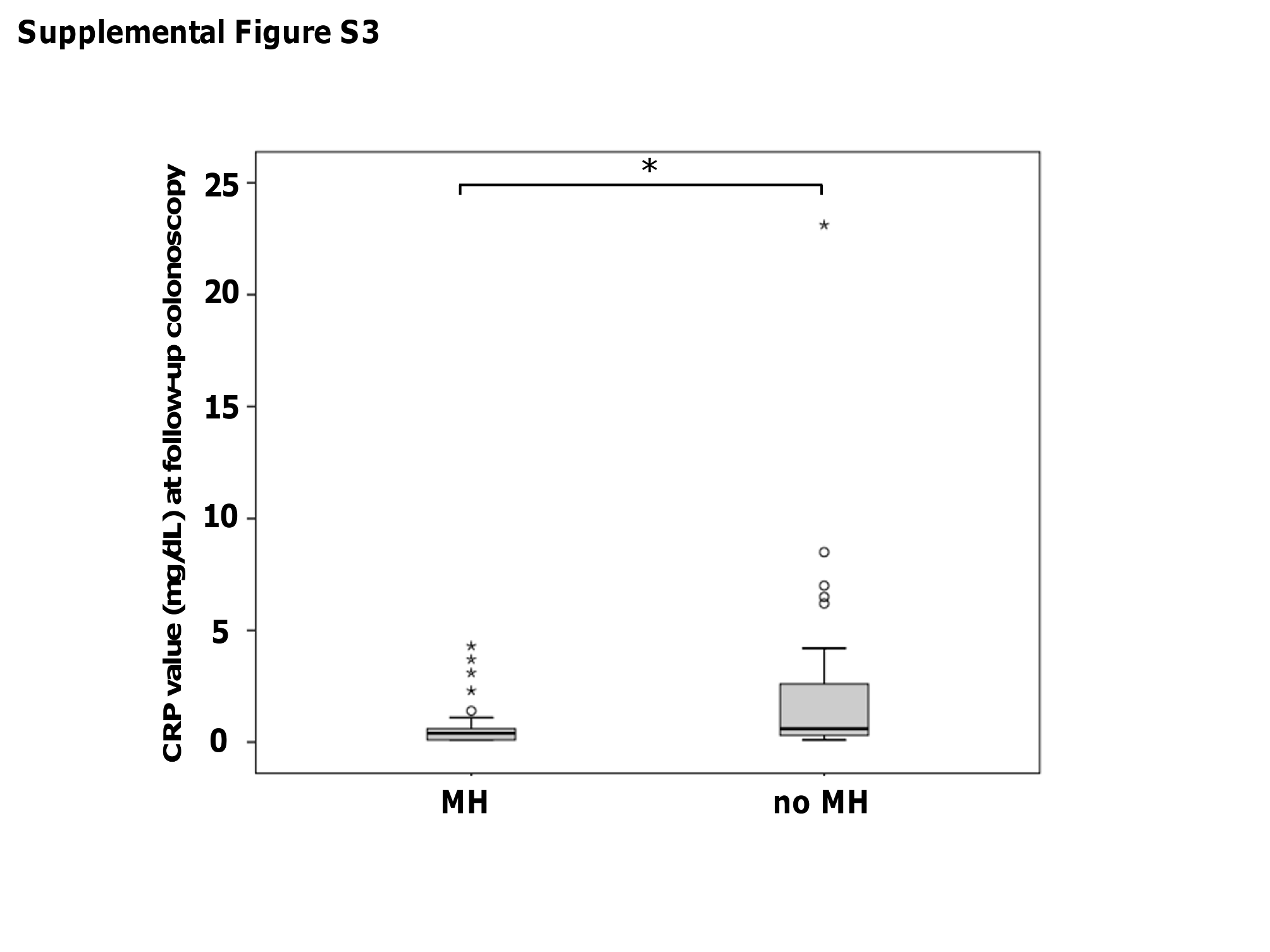

Supplement: Figure S3 — CRP values of CD patients at follow-up colonoscopy (TNF1 group). At follow-up colonoscopy, CRP values were significantly lower in patients with MH compared to patients without MH (*p = 0.01). (TIF) [file pone.0099293.s003.tif]

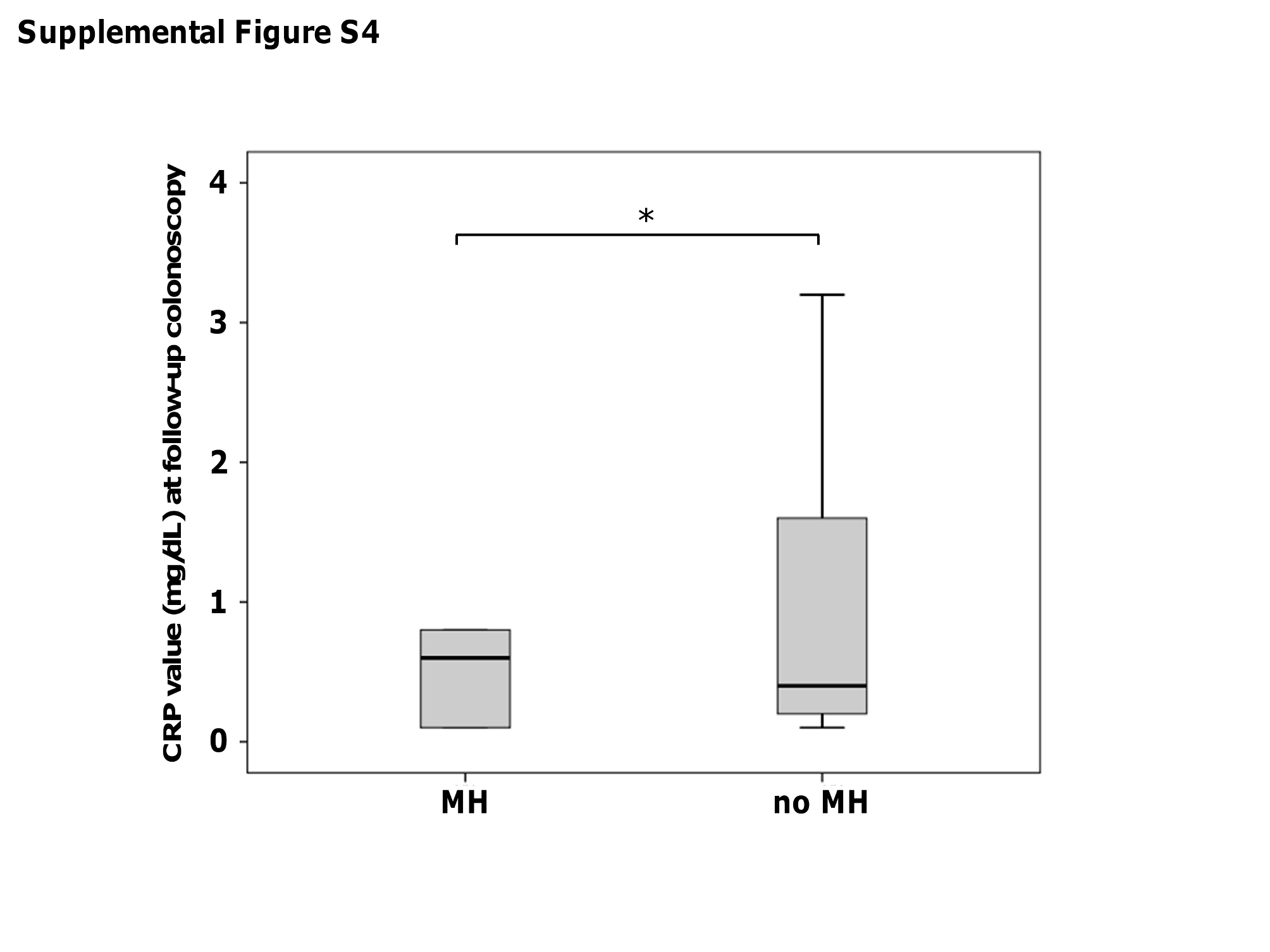

Supplement: Figure S4 — CRP values of CD patients at follow-up colonoscopy (TNF2 group). At follow-up colonoscopy, CRP values were significantly lower in patients with MH compared to patients without MH (*p = 0.01). (TIF) [file pone.0099293.s004.tif]

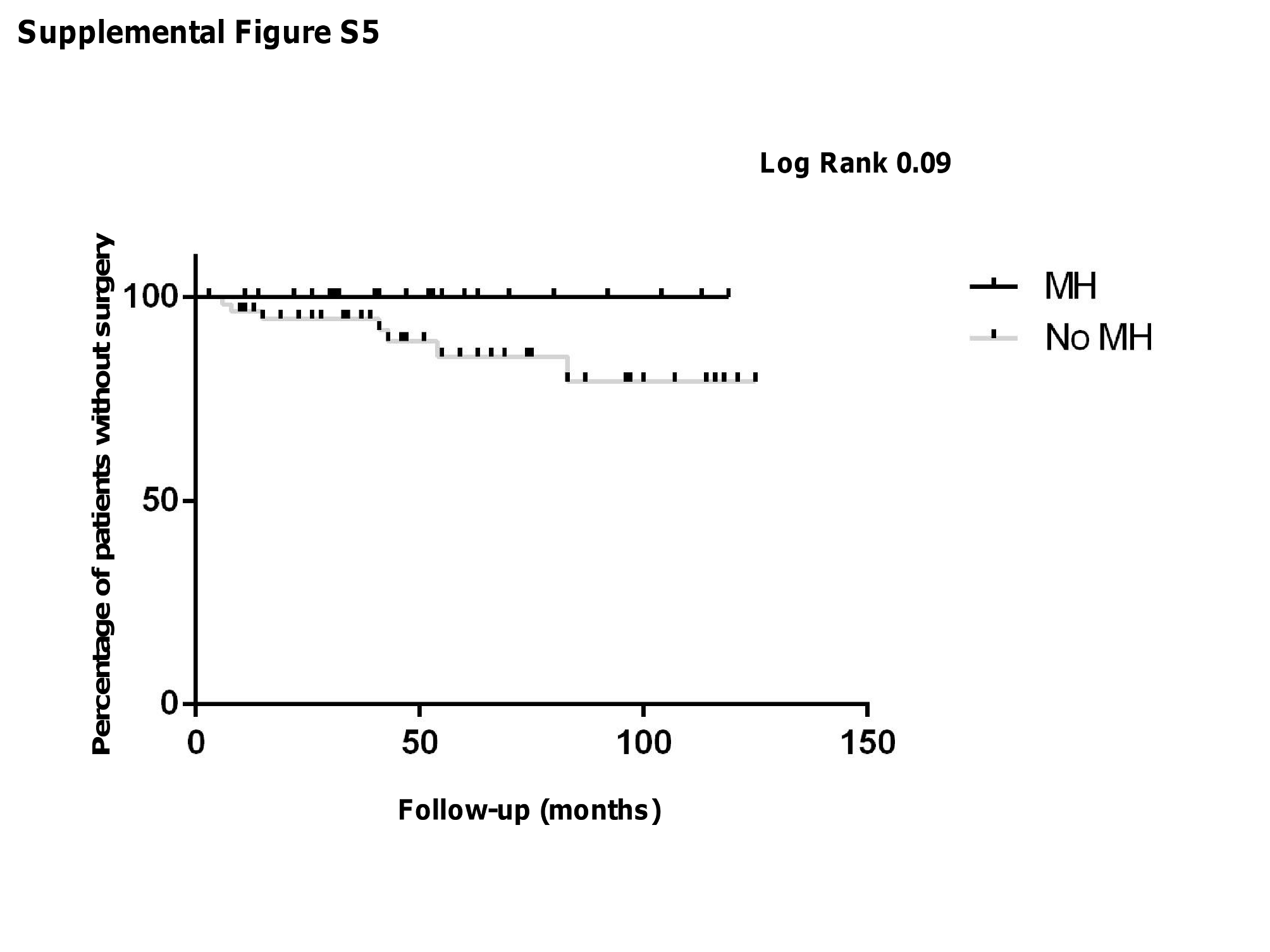

Supplement: Figure S5 — Kaplan-Mayer estimate for surgery-free time intervals in UC patients (TNF1 group) during the follow-up time. During the follow-up time, no patient with MH underwent surgery as compared to 7 patients without MH patients (logrank p = 0.09). (TIF) [file pone.0099293.s005.tif]

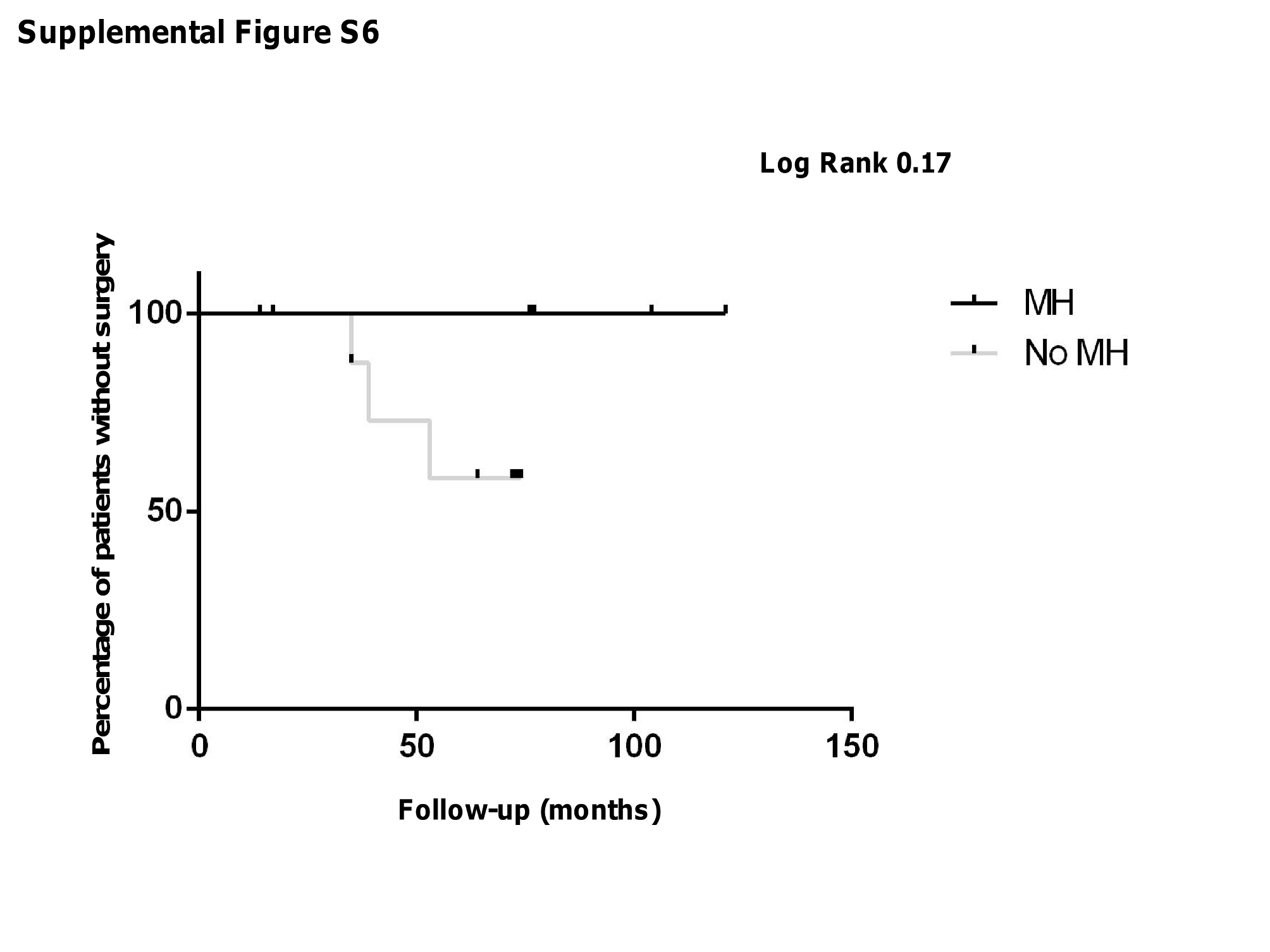

Supplement: Figure S6 — Kaplan-Mayer estimate for surgery-free time intervals in UC patients (TNF2 group) during the follow-up time. During the follow-up time, no patient with MH underwent surgery as compared to 3 patients without MH patients (logrank p = 0.17). (TIF) [file pone.0099293.s006.tif]

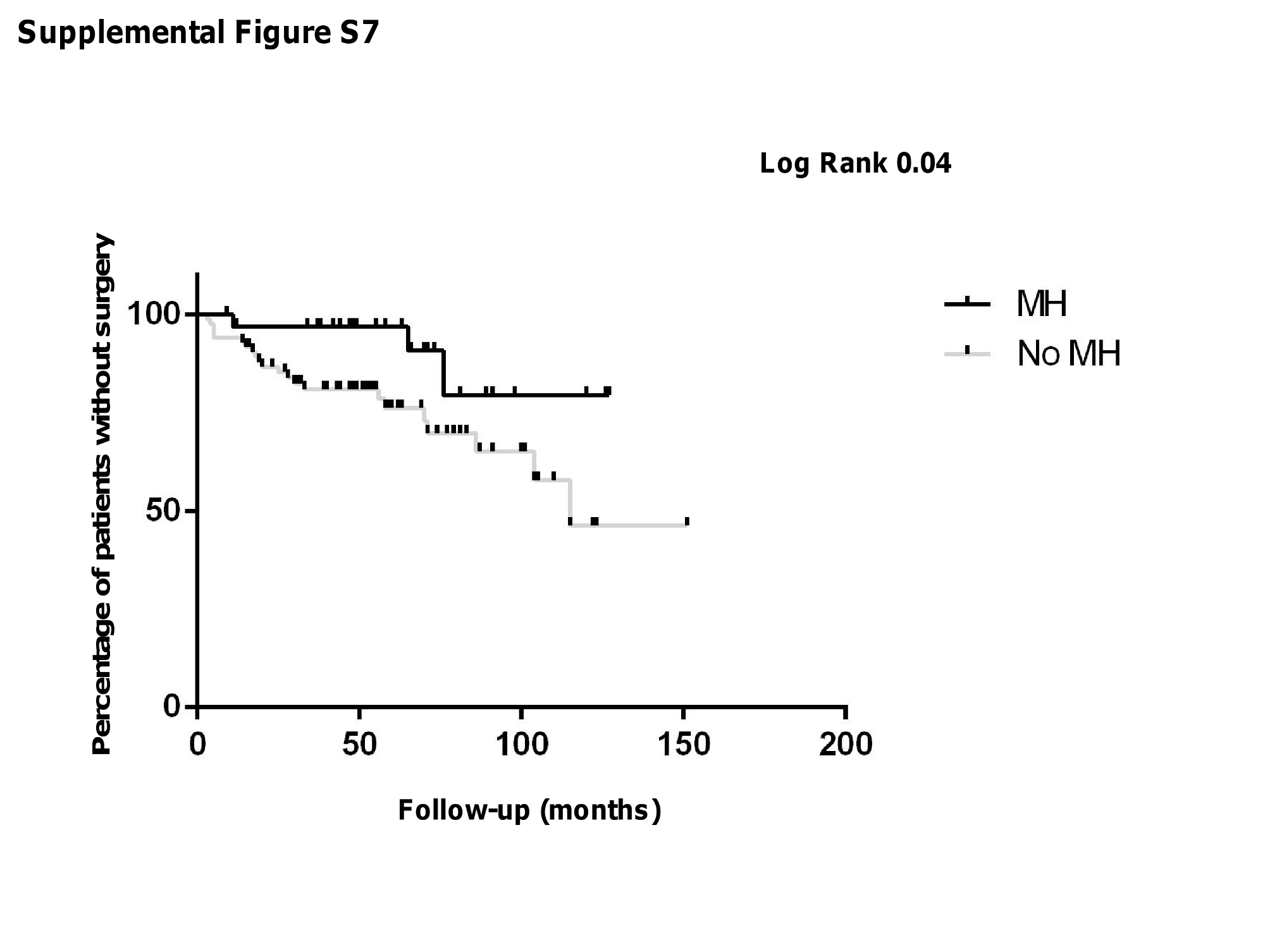

Supplement: Figure S7 — Kaplan-Mayer estimate for surgery-free time intervals in CD patients (TNF1 group) during the follow-up time. During the follow-up time, 3 patients with MH underwent surgery as compared to 24 patients without MH patients (logrank p = 0.04). (TIF) [file pone.0099293.s007.tif]

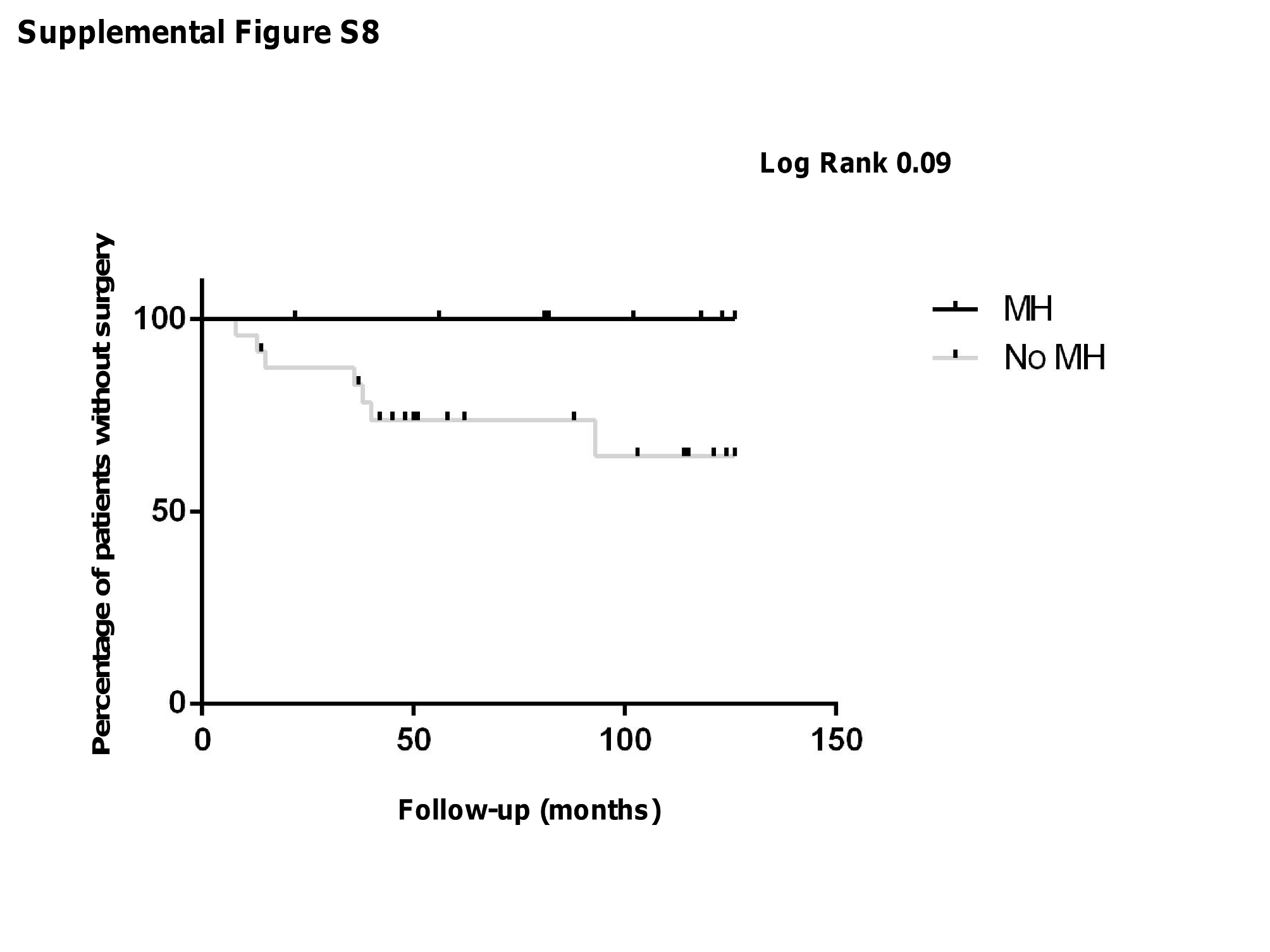

Supplement: Figure S8 — Kaplan-Mayer estimate for surgery-free time intervals in CD patients (TNF2 group) during the follow-up time. During the follow-up time, no patient with MH underwent surgery as compared to 7 patients without MH patients (logrank p = 0.09). (TIF) [file pone.0099293.s008.tif]
