# Supplementary material for: Rate and Predictors of Mucosal Healing in Patients with Inflammatory Bowel Disease Treated with Anti-TNF-Alpha Antibodies
Source: PLoS One. 2014 Jun 16;9(6):e99293. doi: 10.1371/journal.pone.0099293 (PMC4059645; doi:10.1371/journal.pone.0099293)
Supplement: Table S1 — Demographic and clinical characteristics of the UC study cohort (n = 96). (DOC) [file pone.0099293.s009.doc]

**Supplemental Table S1.** Demographic and clinical characteristics of the UC study cohort (n=96).

|  | TNF1 group | TNF2 group |
| --- | --- | --- |
| **Patients** (n=) | 82 | 14 |
| **Median age** (yrs) [Range] | 39 [21;70] | 42 [17;73] |
| **Median age at diagnosis** (yrs) [Range] | 28 [13;63] | 30 [10;68] |
| **Median disease duration** (yrs) [Range] | 10 [0;45] | 7 [2;44] |
| **Female sex** (%) | 42 (51.2) | 7 (50) |
| **Smoker** (%) | 19 (23.2) | 2 (14.2) |
| **Family history of IBD** (%) | 6 (7.3) | 1 (7.1) |
| **Extraintestinal manifestation** (%) | 21 (25.6) | 4 (28.6) |
| **Mean CRP-value at baseline colonoscopy** (mg/dL) [Range] | 2.47 [0.1;14.8] | 1.06 [0.1;3.5] |
| **Mean CRP-value at follow-up colonoscopy** (mg/dL) [Range] | 1.17 [0.1;8.6] | 0.80 [0.1;2.3] |
| **Mean WBC at baseline colonoscopy** (G/L) [Range] | 9.89 [3.9;19.7] | 8.36 [4.4;15.4] |
| **Mean WBC at follow-up colonoscopy (G/L)** [Range] | 7.33 [2.2;14.6] | 6.84 [3.4;19.5] |
| **Thiopurine treatment ever** (%) | 68 (82.9) | 13 (92.9) |
| **Median thiopurine treatment duration** (months) [Range] | 3.5 [0;104] | 25 [18;75] |
| **Infliximab treated patients** (%) | 79 (96.3) | 1 (7.1) |
| **Adalimumab treated patients** (%) | 3 (3.7) | 13 (92.9) |
| **Anti-TNF-alpha antibody and thiopurine treated patients at follow-up** (%) | 12 (14.6) | 2 (14.3) |
| **Median duration infliximab treatment** (months) [Range] | 9 [0;52] | 17 [17;17] |
| **Median duration adalimumab treatment** (months) [Range] | 1 [1;4] | 16 [1;44] |
| **Median thiopurine treatment duration** (months) [Range] | 7 [0;30] | 5 [0;39] |
| **Median time to first anti-TNF-alpha antibody treatment** (years) [Range] | 17.5 [0;88] | 26.5 [6;81] |
| **Median time from baseline to follow-up colonoscopy** (months) [Range] | 9 [0;38] | 6 [0;26] |
| **Patients with surgery till follow-up** (%) | 7 (8.5) | 3 (21.4) |
| **Patients hospitalized till follow-up** (%) | 22 (26.8) | 5 (35.7) |
| **Median follow-up** (months) [Range] | 51.5 [3;125] | 57 [14;103] |
