# Supplementary material for: Rate and Predictors of Mucosal Healing in Patients with Inflammatory Bowel Disease Treated with Anti-TNF-Alpha Antibodies
Source: PLoS One. 2014 Jun 16;9(6):e99293. doi: 10.1371/journal.pone.0099293 (PMC4059645; doi:10.1371/journal.pone.0099293)
Supplement: Table S2 — Demographic and clinical characteristics of the UC TNF1 group (n = 82) regarding MH. (DOC) [file pone.0099293.s010.doc]

**Supplemental Table S2.** Demographic and clinical characteristics of the UC TNF1 group (n=82) regarding MH.

|  | MH | No MH | p-value | OR [95%CI] |
| --- | --- | --- | --- | --- |
| **Patients** (n=) | 23 (28.0) | 59 (72.0) |  |  |
| **Median age** (yrs) [Range] | 38 [21;62] | 40 [22;70] | 0.58 | 1.007 [0.981;1.034] |
| **Median age at diagnosis** (yrs) [Range] | 28 [15;42] | 28.5 [13;63] | 0.03 | 1.039 [1.005;1.074] |
| **Median disease duration** (yrs) [Range] | 11 [0;33] | 9 [0;45] | 0.05 | 0.961 [0.923;1.0] |
| **Female sex** (%) | 11 (47.8) | 31 (52.5) | 0.52 | 1.253, [0.632;2.482] |
| **Smoker** (%) | 5 (21.7) | 14 (23.8) | 0.07 | 0.745 [0.545;1.019] |
| **Family history of IBD** (%) | 5 (21.7) | 6 (10.2) | 0.28 |  |
| **Extraintestinal manifestation** (%) | 7 (30.4) | 14 (23.7) | 0.58 |  |
| **Mean CRP-value at baseline colonoscopy** (mg/dL) [Range] | 2.93 [0.1;14.8] | 2.28 [0.1;12.7] | 0.47 | 0.946 [0.813;1.101] |
| **Mean CRP-value at follow-up colonoscopy** (mg/dL) [Range] | 0.32 [0.1;2.0] | 1.51 [0.1;8.6] | 0.03 | 4.445 [1.133;17.435] |
| **Mean WBC at baseline colonoscopy** (G/L) [Range] | 10.95 [4.7;19.7] | 9.48 [3.9;18.6] | 0.15 | 0.903 [0.785;1.039] |
| **Mean WBC at follow-up colonoscopy (G/L)** [Range] | 6.64 [2.2;14.6] | 7.59 [4.0;13.4] | 0.13 | 1.187[0.949;1.484] |
| **Thiopurine treatment ever** (%) | 18 (78.3) | 50 (84.7) | 0.52 |  |
| **Median thiopurine treatment duration** (months) [Range] | 2 [0;104] | 4 [0;76] | 0.88 | 1.002 [0.978;1.026] |
| **Infliximab treated patients** (%) | 22 (95.7) | 57 (96.6) | 1.0 |  |
| **Adalimumab treated patients** (%) | 1 (4.3) | 2 (3.4) | 1.0 |  |
| **Anti-TNF-alpha antibody and thiopurine treated patients at follow-up** (%) | 4 (17.4) | 8 (13.6) | 0.73 |  |
| **Median duration infliximab treatment** (months) [Range] | 12 [0;52] | 8 [0;41] | 0.31 | 0.979 [0.941;1.019] |
| **Median duration adalimumab treatment** (months) [Range] | 1 [1;1] | 2.5 [1;4] | 1.0 |  |
| **Median time to first anti-TNF-alpha antibody treatment** (years) [Range] | 11 [0;30] | 7 [0;28] | 0.04 | 0.929 [0.868;0.995] |
| **Median time from baseline to follow-up colonoscopy** (months) [Range] | 21 [1;88] | 17 [0;62] | 0.33 | 0.986 [0.957;1.015] |
| **Patients with surgery till follow-up** (%) | 0 | 7 (11.9) | 0.18 |  |
| **Patients hospitalized till follow-up** (%) | 1 (4.3) | 21 (35.6) | 0.005 |  |
| **Median follow-up** (months) [Range] | 52 [11;119] | 51 [3;125] | 0.59 | 1.004 [0.989;1.019] |
