# Supplementary material for: Rate and Predictors of Mucosal Healing in Patients with Inflammatory Bowel Disease Treated with Anti-TNF-Alpha Antibodies
Source: PLoS One. 2014 Jun 16;9(6):e99293. doi: 10.1371/journal.pone.0099293 (PMC4059645; doi:10.1371/journal.pone.0099293)
Supplement: Table S3 — Demographic and clinical characteristics of the UC TNF2 group (n = 14) regarding MH. (DOC) [file pone.0099293.s011.doc]

**Supplemental Table S3.** Demographic and clinical characteristics of the UC TNF2 group (n=14) regarding MH.

|  | MH | No MH | p-value | OR [95%CI] |
| --- | --- | --- | --- | --- |
| **Patients** (n=) | 5 (35.7) | 9 (64.3) |  |  |
| **Median age** (yrs) [Range] | 44 [26;73] | 40 [17;66] | 0.44 | 0.971 [0.899;1.048] |
| **Median age at diagnosis** (yrs) [Range] | 37 [23;68] | 29 [10;50] | 0.12 | 0.916 [0.821;1.023] |
| **Median disease duration** (yrs) [Range] | 5 [3;9] | 10 [2;44] | 0.21 | 1.227 [0.887;1.698] |
| **Female sex** (%) | 2 (40) | 5 (55.6) | 0.57 | 1.875 [0.204;17.269] |
| **Smoker** (%) | 1 (20) | 2 (22.2) | 0.99 |  |
| **Family history of IBD** (%) | 5 (100) | 1 (11.1) | 0.003 |  |
| **Extraintestinal manifestation** (%) | 5 (100) | 4 (44.4) | 0.009 |  |
| **Mean CRP-value at baseline colonoscopy** (mg/dL) [Range] | 0.55 [0.5;0.6] | 1.23 [0.1;3.5] | 0.51 | 2.638 [0.143;46.816] |
| **Mean CRP-value at follow-up colonoscopy** (mg/dL) [Range] | 0.200 [0.1;0.3] | 1.067 [0.1;2.3] | 0.31 | 14.903 [0.076;2926.524] |
| **Mean WBC at baseline colonoscopy** (G/L) [Range] | 8.500 [6.8;10.2] | 8.317 [4.4;15.4] | 0.94 | 0.981 [0.590;1.632] |
| **Mean WBC at follow-up colonoscopy (G/L)** [Range] | 4.675 [4.0;5.9] | 7.811 [3.4;19.5] | 0.25 | 1.724 [0.672;4.422] |
| **Thiopurine treatment ever** (%) | 5 (100) | 8 (88.9) | 1.0 |  |
| **Median thiopurine treatment duration** (months) [Range] | 61.5 [48;75] | 24 [18;25] | 0.05 | 3.0 |
| **Infliximab after adalimumab treated patients (%)** | 1 (20) | 0 | 0.35 |  |
| **Adalimumab after infliximab treated patients (%)** | 4 (80) | 9 | 0.35 |  |
| **Anti-TNF-alpha antibody and thiopurine treated patients (%)** | 1 (20) | 1 (11.1) | 1.0 |  |
| **Median duration anti-TNF-alpha antibody treatment (months)** [Range] | 39 [13;48] | 26 [4;49] | 0.57 | 0.978 [0.912;1.048] |
| **Median duration infliximab treatment** (months) [Range] | 17 [17;17] | 0 | - | - |
| **Median duration adalimumab treatment** (months) [Range] | 18 [6;44] | 16 [1;35] | 0.48 | 0.966 [0.878;1.064] |
| **Median time to first anti-TNF-alpha antibody treatment** (years) [Range] | 4 [0;5] | 8 [0;39] | 0.19 | 1.231 [0.90;1.684] |
| **Median time from baseline to follow-up colonoscopy** (months) [Range] | 27 [17;81] | 26 [6;51] | 0.28 | 0.968 [0.913;1.027] |
| **Median time to first anti-TNF-alpha antibody treatment** (years) [Range] | 7 [1;22] | 5 [0;26] | 0.79 | 0.984 [0.872;1.110] |
| **Surgery till follow-up (%)** | 0 | 3 (33.3) | 0.25 |  |
| **Hospitalization till follow-up (%)** | 0 | 5 (55.6) | 0.08 |  |
| **Median follow-up** (months) [Range] | 73 [17;103] | 50 [14;82] | 0.35 | 0.980 [0.938;1.023] |
