# Supplementary material for: Rate and Predictors of Mucosal Healing in Patients with Inflammatory Bowel Disease Treated with Anti-TNF-Alpha Antibodies
Source: PLoS One. 2014 Jun 16;9(6):e99293. doi: 10.1371/journal.pone.0099293 (PMC4059645; doi:10.1371/journal.pone.0099293)
Supplement: Table S4 — Multivariate analysis for outcome MH in the UC TNF1 group. (DOC) [file pone.0099293.s012.doc]

**Supplemental Table S4. Multivariate analysis for outcome MH in the UC TNF1 group**

|  | p-value | OR [95%CI] |
| --- | --- | --- |
| CRP-value at baseline colonoscopy | 0.099 | 1.50 [0.926;2.428] |
| CRP-value at follow-up colonoscopy | 0.058 | 4.321 [0.950;19.651] |
| WBC at baseline colonoscopy | 0.075 | 0.828 [0.673;1.019] |
| WBC at follow-up colonoscopy | 0.819 | 1.035 [0.773;1.385] |
| Age at diagnosis | 0.966 | 0.942 [0.060;14.747] |
| Age | 0.936 | 1.118 [0.071;17.507] |
| Gender | 0.670 | 1.422 [0.281;7.198] |
| Smoker | 0.513 | 0.781 [0.373;1.636] |
| Duration anti-TNF-alpha antibody treatment | 0.551 | 1.028 [0.939;1.126] |
| Time to first anti-TNF-alpha antibody treatment | 0.196 | 0.805 [0.580;1.118] |
| Time from baseline to follow-up colonoscopy | 0.574 | 0.981 [0.917;1.049] |
