# Supplementary material for: Rate and Predictors of Mucosal Healing in Patients with Inflammatory Bowel Disease Treated with Anti-TNF-Alpha Antibodies
Source: PLoS One. 2014 Jun 16;9(6):e99293. doi: 10.1371/journal.pone.0099293 (PMC4059645; doi:10.1371/journal.pone.0099293)
Supplement: Table S5 — Multivariate analysis for outcome MH in the UC TNF2 group. (DOC) [file pone.0099293.s013.doc]

**Supplemental Table S5.** Multivariate analysis for outcome MH in the UC TNF2 group

|  | p-value | OR [95%CI] |
| --- | --- | --- |
| CRP-value at baseline colonoscopy | 0.523 | 2.695 [0.129;56.434] |
| CRP-value at follow-up colonoscopy | 0.395 | 10.582 [0.046;2441.903] |
| WBC at baseline colonoscopy | 0.926 | 0.977 [0.600;1.591] |
| WBC at follow-up colonoscopy | 0.360 | 2.064 [0.438;9.729] |
| Age at diagnosis | 0.068 | 0.876 [0.760;1.010] |
| Age | 0.382 | 3.132 [0.242;40.448] |
| Gender | 0.448 | 2.028 [0.326;12.595] |
| Duration anti-TNF-alpha antibody treatment | 0.159 | 0.923 [0.825;1.032] |
| Time to first anti-TNF-alpha antibody treatment | 0.422 | 0.895 [0.682;1.174] |
| Time from baseline to follow-up colonoscopy | 0.500 | 0.495 [0.064;3.812] |
| Time from first to second anti-TNF-alpha antibody treatment | 0.422 | 0.895 [0.682;1.174] |
