# Supplementary material for: Rate and Predictors of Mucosal Healing in Patients with Inflammatory Bowel Disease Treated with Anti-TNF-Alpha Antibodies
Source: PLoS One. 2014 Jun 16;9(6):e99293. doi: 10.1371/journal.pone.0099293 (PMC4059645; doi:10.1371/journal.pone.0099293)
Supplement: Table S6 — Demographic and clinical characteristics of the CD study cohort (n = 152). (DOC) [file pone.0099293.s014.doc]

**Supplemental Table S6.** Demographic and clinical characteristics of the CD study cohort (n=152).

|  | TNF1 group | TNF2 group |
| --- | --- | --- |
| **Patients** (n=) | 120 | 32 |
| **Median age** (yrs) [Range] | 34.5 [18;72] | 44 [20;69] |
| **Median age at diagnosis** (yrs) [Range] | 23 [6;63] | 23 [7;57] |
| **Median disease duration** (yrs) [Range] | 9.5 [0;42] | 12.5 [3;39] |
| **Female sex** (%) | 64 (53.3) | 13 (40.6) |
| **Smoker** (%) | 54 (45) | 12 (37.6) |
| **Family history of IBD** (%) | 21 (17.5) | 1 (3.1) |
| **Extraintestinal manifestation** (%) | 59 (49.2) | 20 (62.5) |
| **Mean CRP-value at baseline colonoscopy** (mg/dL) [Range] | 2.36 [0.1;33.8] | 2.84 [0.1;15.4] |
| **Mean CRP-value at follow-up colonoscopy** (mg/dL) [Range] | 1.45 [0.1;23.1] | 1.59 [0.1;10.1] |
| **Mean WBC at baseline colonoscopy** (G/L) [Range] | 8.83 [2.3;23.1] | 10.06 [6.4;23.9] |
| **Mean WBC at follow-up colonoscopy (G/L)** [Range] | 7.61 [1.6;17.3] | 9.03 [2.7;19.3] |
| **Thiopurine treatment ever** (%) | 104 (86.7) | 31 (96.9) |
| **Median thiopurine treatment duration** (months) [Range] | 9.5 [0;211] | 6 [0;237] |
| **Infliximab treated patients** (%) | 106 (88.3) | 3 (9.4) |
| **Adalimumab treated patients** (%) | 14 (11.7) | 29 (90.6) |
| **Anti-TNF-alpha antibody and thiopurine treated patients at follow-up** (%) | 26 (21.6) | 3 (9.4) |
| **Median duration infliximab treatment** (months) [Range] | 12 [0;70] | 19 [1;39] |
| **Median duration adalimumab treatment** (months) [Range] | 11 [2;41] | 10 [0;42] |
| **Median thiopurine treatment duration** (months) [Range] | 7 [0;42] | 8.5 [0;37] |
| **Median time to first anti-TNF-alpha antibody treatment** (years) [Range] | 19.5 [1;111] | 29 [1;75] |
| **Median time from baseline to follow-up colonoscopy** (months) [Range] | 7.1 [0;48] | 8.5 [0;68] |
| **Patients with surgery till follow-up** (%) | 27 (22.5) | 7 (21.9) |
| **Patients hospitalized till follow-up** (%) | 35 (29.5) | 13 (40.6) |
| **Median follow-up** (months) [Range] | 63 [16;127] | 68.5 [9;126] |
