# Supplementary material for: Rate and Predictors of Mucosal Healing in Patients with Inflammatory Bowel Disease Treated with Anti-TNF-Alpha Antibodies
Source: PLoS One. 2014 Jun 16;9(6):e99293. doi: 10.1371/journal.pone.0099293 (PMC4059645; doi:10.1371/journal.pone.0099293)
Supplement: Table S7 — Demographic and clinical characteristics of the CD TNF1 group (n = 120) regarding MH. (DOC) [file pone.0099293.s015.doc]

**Supplemental Table S7.** Demographic and clinical characteristics of the CD TNF1 group (n=120) regarding MH.

|  | MH | No MH | p-value | OR [95%CI] |
| --- | --- | --- | --- | --- |
| **Patients** (n=) | 33 (27.5) | 87 (72.5) |  |  |
| **Median age** (yrs) [Range] | 37 [19;67] | 34 [18;72] | 0.82 | 0.997 [0.968;1.026] |
| **Median age at diagnosis** (yrs) [Range] | 25 [14;48] | 23 [6;63] | 0.44 | 0.987 [0.954;1.021] |
| **Median disease duration** (yrs) [Range] | 7 [0;33] | 10 [0;42] | 0.57 | 1.012 [0.97;1.056] |
| **Female sex** (%) | 20 (60.6) | 44 (50.6) | 0.33 | 0.665 [0.294;1.503] |
| **Smoker** (%) | 19 (57.6) | 35 (40.2) | 0.91 | 0.979 [0.669;1.430] |
| **Family history of IBD** (%) | 3 (9.1) | 18 (20.7) | 0.18 |  |
| **Extraintestinal manifestation** (%) | 16 (48.5) | 43 (49.4) | 1.0 |  |
| **Mean CRP-value at baseline colonoscopy** (mg/dL) [Range] | 2.06 [0.1;11.6] | 2.47 [0.1;33.8] | 0.66 | 1.031[0.897;1.185] |
| **Mean CRP-value at follow-up colonoscopy** (mg/dL) [Range] | 0.75 [0.1;4.3] | 1.72[0.1;23.1] | 0.05 | 1.428 [0.991;2.058] |
| **Mean WBC at baseline colonoscopy** (G/L) [Range] | 8.06 [2.3;16.8] | 9.13 [3.5;23.1] | 0.19 | 1.09 [0.957;1.241] |
| **Mean WBC at follow-up colonoscopy (G/L)** [Range] | 6.45 [1.6;12.9] | 8.07 [3.3;17.3] | 0.007 | 1.294 [1.074;1.560] |
| **Thiopurine treatment ever** (%) | 28 (84.8) | 76 (87.4) | 0.76 |  |
| **Median thiopurine treatment duration** (months) [Range] | 8 [0;72] | 11.5 [0;211] | 0.12 | 1.016 [0.996;1.036] |
| **Infliximab treated patients** (%) | 30 (90.9) | 76 (87.4) | 0.75 |  |
| **Adalimumab treated patients** (%) | 3 (9.1) | 11 (12.6) | 0.75 |  |
| **Anti-TNF-alpha antibody and thiopurine treated patients at follow-up** (%) | 6 (18.2) | 20 (22.9) | 0.62 |  |
| **Median duration infliximab treatment** (months) [Range] | 12 [0;69] | 12.5 [0;70] | 0.81 | 1.003 [0.977;1.030] |
| **Median duration adalimumab treatment** (months) [Range] | 24 [21;41] | 6 [2;25] | 0.13 | 0.714 [0.46;1.11] |
| **Median time to first anti-TNF-alpha antibody treatment** (years) [Range] | 5 [0;30] | 8 [0;42] | 0.24 | 1.029 [0.981;1.079] |
| **Median time from baseline to follow-up colonoscopy** (months) [Range] | 21 [1;71] | 18 [1;111] | 0.81 | 0.998 [0.977;1.018] |
| **Patients with surgery till follow-up** (%) | 3 (9.1) | 24 (27.6) | 0.048 |  |
| **Patients hospitalized till follow-up** (%) | 9 (27.3) | 26 (29.9) | 0.82 |  |
| **Median follow-up** (months) [Range] | 70.5 [38;127] | 58 [16;123] | 0.05 | 0.984 [0.969;1.0] |
