# Supplementary material for: Rate and Predictors of Mucosal Healing in Patients with Inflammatory Bowel Disease Treated with Anti-TNF-Alpha Antibodies
Source: PLoS One. 2014 Jun 16;9(6):e99293. doi: 10.1371/journal.pone.0099293 (PMC4059645; doi:10.1371/journal.pone.0099293)
Supplement: Table S8 — Demographic and clinical characteristics of the CD TNF2 group (n = 32) regarding MH. (DOC) [file pone.0099293.s016.doc]

**Supplemental Table S8.** Demographic and clinical characteristics of the CD TNF2 group (n=32) regarding MH.

|  | MH | No MH | p-value | OR [95%CI] |
| --- | --- | --- | --- | --- |
| **Patients** (n=) | 8 (25.0) | 24 (75.0) |  |  |
| **Median age** (yrs) [Range] | 45 [27;60] | 43.5 [20;69] | 0.32 | 0.979 [0.937;1.022] |
| **Median age at diagnosis** (yrs) [Range] | 23.5 [7;57] | 23 [12;55] | 0.66 | 0.990 [0.948;1.034] |
| **Median disease duration** (yrs) [Range] | 13.5 [3;39] | 12.5 [3;35] | 0.42 | 0.976 [0.921;1.035] |
| **Female sex** (%) | 5 (62.5) | 8 (33.3) | 0.15 | 0.300 [0.092;0.973] |
| **Smoker** (%) | 4 (50) | 8 (33.3) | 0.20 | 0.534 [0.268;1.063] |
| **Family history of IBD** (%) | 1 (12.5) | 0 | 0.25 |  |
| **Extraintestinal manifestation** (%) | 3 (37.5) | 17 (70.8) | 0.12 |  |
| **Mean CRP-value at baseline colonoscopy** (mg/dL) [Range] | 1.34 [0.1;4.9] | 3.39 [0.1;15.4] | 0.23 | 1.309 [0.842;2.037] |
| **Mean CRP-value at follow-up colonoscopy** (mg/dL) [Range] | 0.50 [0.1;0.8] | 1.86 [0.1;10.1] | 0.20 | 2.171 [0.663;7.116] |
| **Mean WBC at baseline colonoscopy** (G/L) [Range] | 9.32 [7.1;12.6] | 10.30[6.4;23.9] | 0.52 | 1.114 [0.802;1.546] |
| **Mean WBC at follow-up colonoscopy (G/L)** [Range] | 10.31 [6.2;19.3] | 8.66 [2.7;18.3] | 0.30 | 0.890 [0.712;1.112] |
| **Thiopurine treatment ever** (%) | 8 (100) | 23 (95.8) | 1.0 |  |
| **Median thiopurine treatment duration** (months) [Range] | 6 [3;36] | 23.5 [0;237] | 0.51 | 1.019 [0.964;1.076] |
| **Infliximab after adalimumab treated patients (%)** | 0 | 3 | 0.55 |  |
| **Adalimumab after infliximab treated patients (%)** | 8 | 21 | 0.55 |  |
| **Anti-TNF-alpha antibody and thiopurine treated patients (%)** | 0 | 3 | 0.55 |  |
| **Median duration anti-TNF-alpha antibody treatment (months)** [Range] | 14.5 [9;95] | 29.5 [0;68] | 0.76 | 1.006 [0.969;1.045 |
| **Median duration infliximab treatment** (months) [Range] | 0 | 19 [1;39] | - | - |
| **Median duration adalimumab treatment** (months) [Range] | 9.5 [2;40] | 10 [0;42] | 0.68 | 0.987 [0.928;1.05] |
| **Median time to first anti-TNF-alpha antibody treatment** (years) [Range] | 9 [1;37] | 8.5 [0;34] | 0.59 | 0.979 [0.906;1.058] |
| **Median time from baseline to follow-up colonoscopy** (months) [Range] | 18.5 [12;68] | 31 [1;75] | 0.63 | 1.010 [0.968;1.054] |
| **Median time to first anti-TNF-alpha antibody treatment** (years) [Range] | 5 [1;68] | 12 [0;56] | 0.55 | 1.016 [0.964;1.071] |
| **Surgery till follow-up (%)** | 0 | 7 (29.2) | 0.14 |  |
| **Hospitalization till follow-up (%)** | 2 (25.0) | 11 (45.2) | 0.42 |  |
| **Median follow-up** (months) [Range] | 87.5 [14;123] | 61.5 [9;126] | 0.57 | 0.993 [0.970;1.017] |
