# Supplementary material for: Rate and Predictors of Mucosal Healing in Patients with Inflammatory Bowel Disease Treated with Anti-TNF-Alpha Antibodies
Source: PLoS One. 2014 Jun 16;9(6):e99293. doi: 10.1371/journal.pone.0099293 (PMC4059645; doi:10.1371/journal.pone.0099293)
Supplement: Table S9 — Multivariate analysis for outcome MH in the MC TNF1 group. (DOC) [file pone.0099293.s017.doc]

**Supplemental Table S9. Multivariate analysis for outcome MH in the MC TNF1 group.**

|  | p-value | OR [95%CI] |
| --- | --- | --- |
| CRP-value at baseline colonoscopy | 0.729 | 0.961 [0.766;1.205] |
| CRP-value at follow-up colonoscopy | 0.079 | 1.496 [0.955;2.342] |
| WBC at baseline colonoscopy | 0.321 | 1.094 [0.917;1.305] |
| WBC at follow-up colonoscopy | 0.036 | 1.263 [1.015;1.572] |
| Age at diagnosis | 0.745 | 1.405 [0.181;10.936] |
| Age | 0.756 | 0.722 [0.092;5.635] |
| Gender | 0.090 | 0.410 [0.146;1.150] |
| Smoker | 0.542 | 0.862 [0.535;1.388] |
| Duration anti-TNF-alpha antibody treatment | 0.868 | 0.995 [0.942;1.052] |
| Time to first anti-TNF-alpha antibody treatment | 0.018 | 1.329 [1.051;1.681] |
| Time from baseline to follow-up colonoscopy | 0.896 | 1.003 [0.956;1.053] |
