# Supplementary material for: Rate and Predictors of Mucosal Healing in Patients with Inflammatory Bowel Disease Treated with Anti-TNF-Alpha Antibodies
Source: PLoS One. 2014 Jun 16;9(6):e99293. doi: 10.1371/journal.pone.0099293 (PMC4059645; doi:10.1371/journal.pone.0099293)
Supplement: Table S10 — Multivariate analysis for outcome MH in the MC TNF2 group. (DOC) [file pone.0099293.s018.doc]

**Supplemental Table S10.** Multivariate analysis for outcome MH in the MC TNF2 group.

|  | p-value | OR [95%CI] |
| --- | --- | --- |
| CRP-value at baseline colonoscopy | 0.342 | 1.274 [0.774;2.097] |
| CRP-value at follow-up colonoscopy | 0.280 | 2.122 [0.542;8.316] |
| WBC at baseline colonoscopy | 0.403 | 1.190 [0.792;1.787] |
| WBC at follow-up colonoscopy | 0.287 | 0.863 [0.658;1.132] |
| Age at diagnosis | 0.357 | 0.532 [0.139;2.039] |
| Age | 0.389 | 1.767 [0.484;6.450] |
| Gender | 0.057 | 0.017 [0.000;1.131] |
| Smoker | 0.066 | 0.201 [0.036;1.114] |
| Duration anti-TNF-alpha antibody treatment | 0.129 | 0.827 [0.648;1.057] |
| Time to first anti-TNF-alpha antibody treatment | 0.351 | 0.553 [0.159;1.923] |
| Time from baseline to follow-up colonoscopy | 0.079 | 1.196 [0.979;1.462] |
| Time from first to second anti-TNF-alpha antibody treatment | 0.980 | 0.998 [0.882;1.131] |
